# Shifts in Assembly Rules and Loss of Zooplankton Functional Diversity Across Hypereutrophic Fishponds

**DOI:** 10.1111/ele.70289

**Published:** 2025-12-11

**Authors:** Cihelio A. Amorim, Martin J. Kainz

**Affiliations:** ^1^ WasserCluster Lunz—Biologische Station GmbH Lunz am See Austria; ^2^ Research Lab for Aquatic Ecosystem Research and Health University for Continuing Education—Danube University Krems Krems an der Donau Austria

**Keywords:** assembly rules, competition, eutrophication, functional homogenization, habitat filtering, limiting similarity, pond biodiversity, stress‐dominance hypothesis, threats to biodiversity, trait convergence

## Abstract

Freshwater species are facing massive declines, often driven by eutrophication. Identifying which facets of biodiversity are sensitive is crucial, as species loss does not always translate to reduced ecosystem functioning and functional diversity. We examined how assembly rules shape zooplankton functional diversity in hypereutrophic fishponds. Higher eutrophication was hypothesised to cause functional homogenization through reduced functional diversity, habitat filtering, and trait convergence. Higher eutrophication indeed reduced functional diversity metrics, whereas species richness was kept stable. Functional richness, dispersion, and dissimilarity shifted from limiting similarity, where niche partitioning and competition shape community structure, to random (incidence data) and habitat filtering (biomass) with increasing eutrophication. Functional divergence transitioned from random to habitat filtering, whereas redundancy increased at higher trophic states. Trait convergence was the dominant process, with the environment selecting species with similar traits. Biodiversity assessments and managers should consider how functional diversity and ecosystem functions respond to anthropogenic and environmental changes.

## Graphical abstract

Functional diversity of zooplankton declined sharply with increasing eutrophication in hypereutrophic fishponds, despite stable species richness. Community assembly shifted from competition to habitat filtering, leading to functional homogenization (redundancy) and altered interactions among functional guilds. Ponds with total phosphorus below 300 μg/L maintained higher taxonomic diversity with early signs of disrupted functional diversity, ecosystem functioning, and stability.

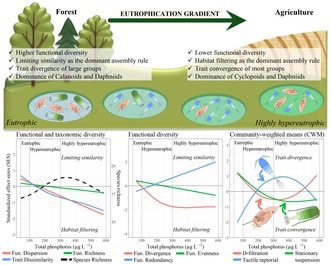

## 1 Introduction

Freshwater biodiversity is facing major threats, often attributed to pollution and eutrophication (Dudgeon 2019; Sayer et al. 2025). This biodiversity decline has severe implications for ecosystem functioning and services (Cardinale et al. 2012). Excessive nutrient enrichment (e.g., nitrogen and phosphorus), intensified by agriculture, growing population, and climate change, is expected to increase further during the 21st century (Sinha et al. 2017). These processes create severe damage to freshwater ecosystems and biota, such as algal blooms, oxygen depletion, reduced water transparency, and loss of biodiversity (Smith and Schindler 2009; Amorim and Moura 2021). Equally important, several ecosystem services are affected, including food security, warning for needed reductions in external loadings of nitrogen and phosphorus (Jeppesen et al. 2025).

Ponds and small water bodies harbour high biodiversity and serve as critical sites for biogeochemical cycling and food web interactions (Céréghino et al. 2014). Even highly eutrophic fishponds may support high taxonomic richness (Wezel et al. 2014), which is less impacted by eutrophication (Rosset et al. 2014). Greater biodiversity improves ecosystem functioning and stability (Tilman et al. 2006; Pennekamp et al. 2018). However, the diversity–stability relationship is multifaceted, involving complex interactions between species richness, functional traits, and phylogenetic diversity (Craven et al. 2018). Although most studies have focused on taxonomic diversity (e.g., Cardinale et al. 2012), evidence suggests that phylogenetic (Cadotte et al. 2012) and functional (Van Der Plas 2019) diversity metrics are better predictors of ecosystem functioning.

The mechanisms underlying the assembly of biological communities have been a central focus in ecology for many decades (e.g., Hutchinson 1961). Assembly rules determine the mechanisms that drive species distributions, coexistence, and community composition (Götzenberger et al. 2012). They predict the critical role of traits and environmental filters in shaping communities, as only species with traits compatible with the habitat's conditions will survive and thrive (Weiher and Keddy 1995). The main approaches to studying ecological assembly rules include (a) species co‐occurrence, through competitive exclusion; (b) niche limitation, where limited niches restrict coexistence; (c) guild proportionality, where competition or environmental filters act differently in the guilds; and (d) limiting similarity, where traits become dissimilar to avoid competitive exclusion (Götzenberger et al. 2012). Trait divergence, a tool for detecting limiting similarity, predicts that coexisting species possess different traits to better exploit available niches, whereas trait convergence, applied to identify habitat filtering, predicts that species are functionally redundant as the restricted number of niches selects for a specific set of traits (Grime 2006).

Assembly rules have largely been developed and implemented for plant and bird assemblages (Götzenberger et al. 2012), whereas attempts to study the assembly rules of plankton communities using trait‐based approaches remain relatively scarce (e.g., Amorim and Moura 2022; Borics et al. 2020; Klais et al. 2017), with even fewer efforts focusing on zooplankton (e.g., Vogt et al. 2013). Besides being key consumers and conveyors of dietary energy within the planktonic food web, zooplankton possess diverse functional attributes and ecological strategies (Litchman et al. 2013). Despite their fundamental role in trait‐based ecology, there have been contrasting results on how eutrophication influences zooplankton assembly rules. For example, research has shown a decrease in the functional diversity (FD) of zooplankton under eutrophic conditions (e.g., Moody and Wilkinson 2019; Fernández‐Aláez et al. 2025), whereas FD in Canadian lakes correlated positively with lake productivity (Vogt et al. 2013). These results point to the relevance of exploring zooplankton assembly rules in the face of the alarming rates of extinction driven by eutrophication (Sayer et al. 2025).

**FIGURE 1:**
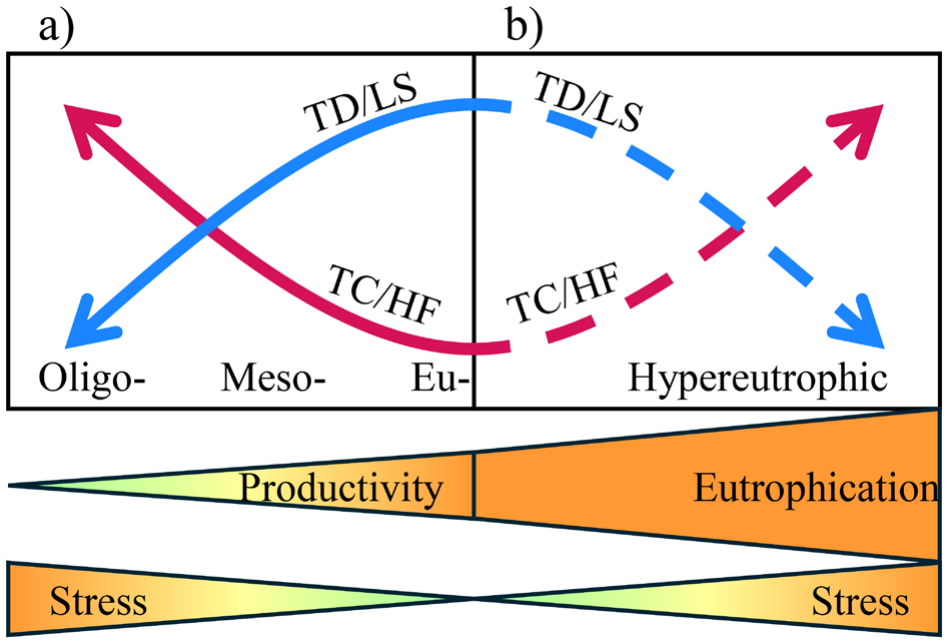
Conceptual diagram showing the predictions of the “stress‐dominance hypothesis” (a) and the hypothesis tested herein (b). The stress‐dominance hypothesis posits an increased role of trait convergence (TC) and habitat filtering (HF) in harsh, less productive environments, alongside a reduction in trait divergence (TD) and limiting similarity (LS) (a). Extreme productivity levels (here, hypereutrophic environments) may also be stressful for aquatic communities (e.g., zooplankton), resulting in greater trait convergence and habitat filtering, while decreasing trait divergence and limiting similarity (b).

Productive terrestrial ecosystems are known to promote trait divergence, whereas nutrient‐limited environments are stressful for the biotic communities and lead to trait convergence (“stress‐dominance hypothesis”) (Coyle et al. 2014) (Figure 1a). Phytoplankton communities may exhibit both trait convergence and divergence, and increased productivity (e.g., eutrophication) drives trait divergence, supporting the stress‐dominance hypothesis (Borics et al. 2020). Despite this, most studies presume that productive environments benefit communities because of higher nutrient and food availability, often overlooking the detrimental effects of extreme eutrophication on aquatic systems (Jeppesen et al. 2025).

In this study, we investigated how pond eutrophication influences FD and zooplankton assembly rules. We answered the following questions: (a) how does eutrophication affect FD and functional guilds; and (b) which assembly rule (e.g., habitat filtering or limiting similarity) governs zooplankton communities under hypereutrophic conditions? We hypothesised that higher eutrophication (i.e., harsher environments) leads to functional homogenization through trait convergence, with habitat filtering consequently becoming the dominant assembly process (Figure 1b). To the best of our knowledge, this is the first study that documents non‐random assembly processes in zooplankton assemblages. We have advanced research on community assembly rules by addressing classic gaps that have persisted in ecological studies (Weiher and Keddy 1995; Shinohara et al. 2023). We have described and explained assembly rules, focusing on how functional traits and species diversity control patterns along environmental gradients, such as eutrophication.

## 2 Materials and Methods

### 2.1 Study Sites

The study was conducted in nine shallow fishponds (maximum depth < 3 m) located in the Waldviertel region of Lower Austria, Austria, from March to September 2024. The surroundings of the ponds consist primarily of forests, agricultural lands, and urban areas (Figure S1). The ponds are primarily stocked with common carp ( *Cyprinus carpio* Linnaeus, 1758) and occasionally with whitefish (*Coregonus* sp.) and tench ( *Tinca tinca* Linnaeus, 1758). The fish are harvested in autumn by draining the ponds and restocked in late winter or early spring. In 2024, the ponds were similarly stocked with 3–4‐year‐old carp at a density of 350–450 carp ha^−1^. Fish diets were supplemented with fish feed.

### 2.2 Sampling and Analysis

Pond sampling was carried out monthly from March (after ice melt) to September 2024 (before pond drainage) to assess basic physicochemical parameters, as well as zooplankton diversity and biomass, covering the entire zooplankton growing season. Water temperature, pH, and electrical conductivity were measured in situ with a multiparameter probe (OTT Hydrolab HL4, Berlin, Germany), and water transparency was estimated using a Secchi disk. Water samples were collected with a Schindler trap (5 L). Total phosphorus (TP) and soluble reactive phosphorus (SRP) were determined following Hansen and Koroleff (1999). Nitrate (NO_3_), nitrite (NO_2_), and ammonia (NH_4_) were analysed on a continuous‐flow analyser (Alliance Instruments GmbH, Flowsys EC, Salzburg, Austria) and dissolved organic carbon (DOC) on an elemental analyser (Thermo Fischer Scientific, Flash 2000—HT Plus, Waltham, USA). Dissolved inorganic nitrogen (DIN) was calculated as the sum of NO_3_, NO_2_, and NH_4_.

Qualitative samples were collected from March to September (*n* = 63), whereas quantitative samples were collected from July to September (*n* = 27). The entire qualitative sample was checked for the composition and species richness of rotifers, cladocerans, and copepods, identified to species level. Quantitative integrated zooplankton samples (20 L) were collected with a Schindler trap, pooled from different depths, filtered through a plankton net (55 μm mesh size), and immediately preserved with Ethanol (96%). At least 300 individuals of the most abundant and 50 of the less abundant species were counted (modified from Mack et al. 2012) in at least three 2.5‐mL aliquots under an inverted microscope. Zooplankton density (ind. L^−1^) was converted to biomass (μg L^−1^) using length‐dry weight regressions (e.g., Dumont et al. 1975; Ejsmont‐Karabin 1998).

Species richness was calculated as the total number of species per sample in the incidence matrix, and Simpson's diversity was calculated in the R package “vegan” using the biomass matrix. Four functional traits were estimated for each species on the basis of their morphology or using data from the literature (e.g., Barnett et al. 2007; Obertegger and Flaim 2015): size classes (morphological; ordinal), feeding type (behavioural and physiological; categorical), trophic group (behavioural and physiological; categorical), and habitat (behavioural; categorical). These traits are translated into resource acquisition, growth, reproduction, and survival ecosystem functions (Martini et al. 2021).

### 2.3 Functional Diversity, Community‐Weighted Means, and Null Models

Functional trait, incidence, and biomass matrices of pond zooplankton communities were used to calculate FD indices. Biomass was transformed using the Hellinger transformation, and the trait matrices were converted into a Gower's dissimilarity matrix (Podani 1999). Functional richness (FRic), evenness (FEve), divergence (FDiv), dispersion (FDis), and dissimilarity (RaoQ: Rao's quadratic entropy) were calculated in the R package “FD” (Laliberte and Legendre 2010). Functional redundancy was computed by dividing the trait dissimilarity (RaoQ) by Simpson's index (D) (FRed = 1–(RaoQ/D)) (Ricotta et al. 2016). We calculated FRic, FDis, and RaoQ using the incidence data because those metrics depend solely on the convex hull volume in trait space (Laliberte and Legendre 2010).

Community‐weighted means (CWM) were calculated as the relative abundance of all species possessing a specific trait using the biomass matrix. The CWM was determined for size classes (< 200, 200–600, and > 600 μm), feeding types (microphagous (Rotifera), raptorial rotifers (Rotifera), stationary suspension (Calanoida), tactile‐raptorial (Cyclopoida, Harpacticoida, and Leptodoridae), D‐ (Daphniidae), B‐ (Bosminidae), C‐ (Chydoridae), and S‐ (Sididae) filtration types), trophic groups (herbivorous, omnivorous, carnivorous, and detritivorous), and habitat (littoral and pelagic).

Trait convergence and divergence, the assembly rules, habitat filtering, and limiting similarity were estimated by comparing observed values of CWM and FD indices with random expectations generated by null models (Gotelli 2000). For that, we created 1000 randomly assembled communities through the randomisation of species abundances in all communities, keeping species frequencies and total abundances constant, while allowing for changes in species richness, using the “*c0*” (for the incidence matrix) and “*c0_samp*” (for the biomass matrix) algorithms in the “vegan” R package (Gotelli 2000). Standardised effect sizes (SES) were calculated by dividing the difference between observed and random mean values by the standard deviation of null models (de Bello 2012). For CWM, positive SES values indicate trait divergence, and negative values indicate trait convergence (de Bello 2012). For FD, positive SES values suggest limiting similarity, and negative SES values indicate habitat filtering (Mouchet et al. 2010). Given the strong linear dependence of SES on observed FD (de Bello 2012), shifts in SES values may be interpreted as mirroring the patterns of observed FD metrics.

### 2.4 Data Analysis

All statistical analyses were conducted using R 4.5.1, with the significance level set at *p* < 0.05. The associated datasets and R code used in this study are available in Figshare at https://doi.org/10.6084/m9.figshare.29400842 (Amorim and Kainz 2025). A principal component analysis (PCA) was applied to summarise environmental variables among fishponds, followed by a PERMANOVA to test for multivariate differences across trophic states (“vegan” and “pairwiseAdonis” packages).

To account for non‐linear patterns, generalised additive mixed models (GAMM, package “mgcv”) (Wood 2017) were employed to predict the impacts of eutrophication (log‐transformed TP) on the response variables. Pond location and sampling time (month) were treated as random factors to account for possible spatial and temporal autocorrelation. Fish predation was not a contributing random factor, as all the ponds had similar stocks. Appropriate families and link functions were chosen depending on the distributions of the response variables (QQ plots and histogram of residuals) and Akaike Information Criterion (AIC) values. To represent different levels of eutrophication, the plots show three trophic state divisions: eutrophic (40–100 μg L^−1^), hypereutrophic (100–300 μg L^−1^), and highly hypereutrophic (> 300 μg L^−1^) levels (adapted from Nürnberg 1996 and Meyer et al. 2025). This splitting is intended to highlight variation in the response variables along the eutrophication gradient rather than between levels. One‐sample *t*‐tests or Wilcoxon signed‐rank tests were used to determine whether SES values differed from zero for the entire dataset or for trophic states, depending on data normality assessed with Shapiro tests.

Two piecewise structural equation models (pSEM, package “piecewiseSEM”) (Lefcheck 2016) were fitted using generalised linear models to test for direct and indirect impacts of eutrophication (log‐transformed TP), functional traits (richness or CWM), and taxonomic diversity (log‐transformed species richness and Simpson's index) on FD metrics (FRic, FDiv, FEve, FRed, and the latent variable FD, calculated by averaging FDis and RaoQ because of their strong correlation: *r* = 0.97). The first model used the incidence matrix and species richness of relevant functional traits, whereas the second used the biomass matrix and CWM of the traits. Goodness‐of‐fit was evaluated through Fisher's *C* statistic and its *p*‐value. The AIC was adjusted for the low sample size using the *C* Information Criterion (AIC_*C*_). Correlation among explanatory variables was assessed using Spearman's test. Robust bootstrapping was employed to quantify uncertainty in path coefficients and to reduce Type I error arising from model choice and sample size. The data were resampled with replacement 999 times, and the pSEM model was reestimated for each bootstrap. Paths were considered truly significant if their 95% bootstrap CIs did not include zero (Thulin 2024). A Monte Carlo power analysis estimated the adequacy of sample size for the pSEM model using the “mvrnorm” function from the “MASS” package. 999 datasets were simulated with preserved empirical means, variances, and correlations. The full pSEM was refitted, and statistical power for each path was estimated using the proportion of simulations in which each path's effect was significant (Thulin 2024).

## 3 Results

### 3.1 Environmental Conditions and Zooplankton Community Responses to Eutrophication

All fishponds were classified as hypereutrophic (except Asang pond), with average total phosphorus (TP) concentrations exceeding 100 μg L^−1^. Großer Harabruck and Pilz fishponds were the most hypereutrophic. The ponds had acidic to neutral pH, with low electrical conductivity and SRP levels. Temperature, TP, DOC, and electrical conductivity increased from March to August, whereas DIN and transparency showed the opposite trend. Aside from TP, no other variable showed consistent differences among the ponds (Figure S2). Multivariate differences in physicochemical parameters were observed across the three trophic state levels (PERMANOVA, *F* = 9.63, *p* < 0.001; Figure S3).

**FIGURE 2:**
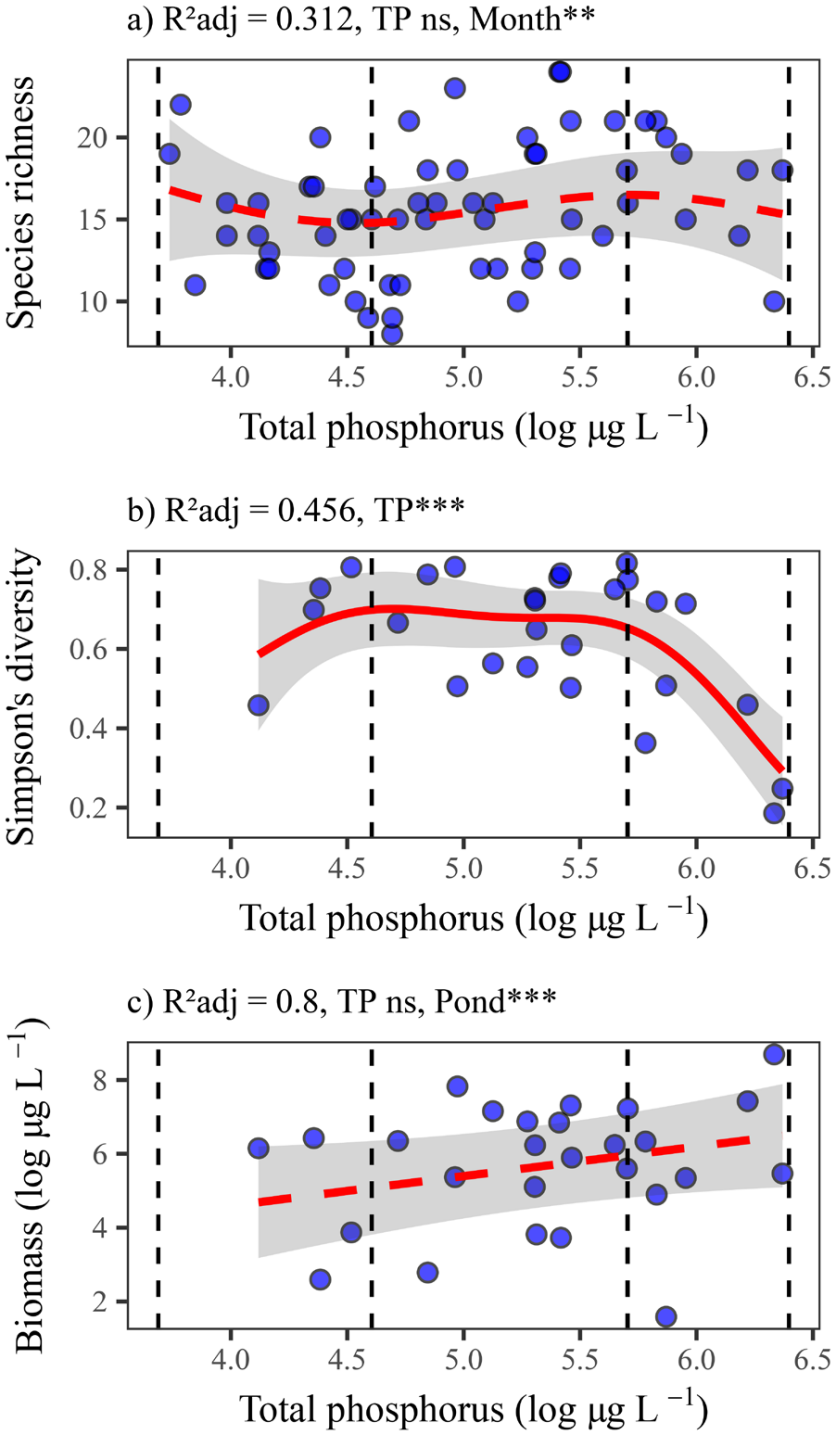
Eutrophication reduced Simpson's diversity despite stable species richness and biomass. Effects of eutrophication (log‐transformed total phosphorus, TP) on species richness (a), Simpson's diversity (b), and total biomass (c, log‐transformed biomass) of zooplankton in the studied fishponds. Models were fitted using generalised additive mixed models (GAMM), accounting for spatial and temporal autocorrelation by adding pond location and sampling time (month) as random factors. Solid red lines represent the significant effects of TP. Vertical dashed lines separate the TP gradient into eutrophic (40–100 μg L^−1^), hypereutrophic (100–300 μg L^−1^), and highly hypereutrophic (> 300 μg L^−1^) levels. ns non‐significant, **p < 0.01, ***p < 0.001.

A total of 59 zooplankton species were identified, distributed in rotifers (30 spp.), cladocerans (19 spp.), calanoid copepods (2 spp.), cyclopoid copepods (7 spp.), and harpacticoid copepods (1 spp.). The calanoid copepod *Acanthodiaptomus denticornis* (Wierzejski 1887) was dominant in Asang fishpond (relative abundance > 50%); the cladoceran *Daphnia galeata* Sars, 1863 was dominant in Asang and Großer Harabruck fishponds; copepod nauplii and the cladoceran *Daphnia curvirostris* Eylmann, 1887 were dominant in Pilz pond; the cyclopoid copepod *Acanthocyclops americanus* (Marsh, 1892) was dominant in Gebharts, Großer Harabruck, Schandachen, and Winkelauer fishponds; and the cyclopoid copepod *Mesocyclops leuckarti* (Claus, 1857) was dominant in Haslawer fishpond. Species richness was not influenced by total phosphorus but showed significant differences across months (random effects). Simpson's diversity index was significantly lower under highly hypereutrophic conditions. Lastly, total biomass did not respond to the eutrophication gradient, but it was influenced by pond identity (random effects) (Figure 2; Table S2).

### 3.2 Functional Diversity and Assembly Rules: Shifts From Limiting Similarity to Habitat Filtering

**FIGURE 3:**
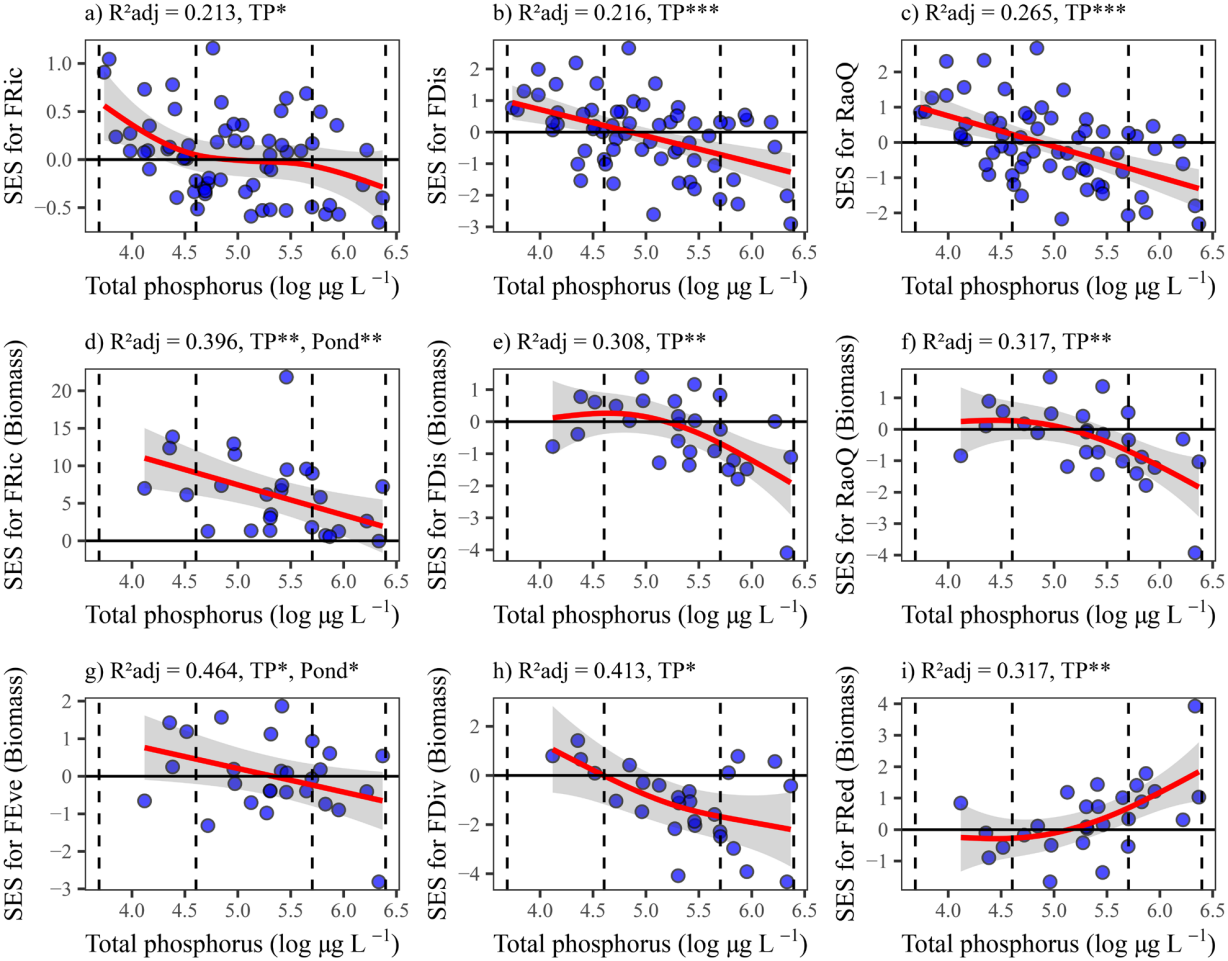
Sharp declines in functional diversity and shifts in assembly rules driven by eutrophication. Changes in standardised effect sizes (SES) from the incidence data: functional richness (FRic, a), dispersion (FDis, b), and trait dissimilarity (RaoQ, c); and from the biomass data: functional richness (FRic, d), dispersion (FDis, e), trait dissimilarity (RaoQ, f), evenness (FEve, g), divergence (FDiv, h), and redundancy (FRed, i) along the eutrophication gradient (log‐transformed total phosphorus, TP). Values below zero represent habitat filtering, and values above zero indicate limiting similarity. Models were fitted using generalised additive mixed models (GAMM), accounting for spatial and temporal autocorrelation by adding pond location and sampling time (month) as random factors. Solid red lines represent the significant effects of TP. Vertical dashed lines separate the TP gradient into eutrophic (40–100 μg L^−1^), hypereutrophic (100–300 μg L^−1^), and highly hypereutrophic (> 300 μg L^−1^) levels. ns non‐significant, *p < 0.05, **p < 0.01, ***p < 0.001.

FRic, FDis, RaoQ (from both incidence and biomass matrices), FEve, and FDiv significantly decreased with eutrophication. FRic and FEve were further influenced by ponds (random effects). FRed was high (> 0.7) but did not significantly respond to the eutrophication gradient (Figure S4; Table S1). SES values comparing observed and expected values under null models for the incidence data revealed major shifts from limiting similarity at eutrophic conditions to random assembly patterns for FRic, FDis, and RaoQ at hypereutrophic and highly hypereutrophic states, with significant declines along the eutrophication gradient. Biomass‐based SES for FRic declined with increasing eutrophication but remained positive, approaching random assembly at higher levels of eutrophication. FDiv patterns were generally driven by habitat filtering processes (negative SES), mainly at hypereutrophic levels, whereas SES for FDis and RaoQ shifted from random to habitat filtering at highly hypereutrophic states, facing strong negative effects of TP. SES for FEve was governed by random processes but declined with TP; meanwhile, SES for FRed increased with eutrophication, reaching values higher than expected under null models at highly hypereutrophic ponds (Figure 3; Tables S2 and S3).

### 3.3 Impacts of Eutrophication on Trait Convergence and Divergence

The zooplankton community in the studied fishponds mainly consisted of organisms larger than 600 μm, with a median relative abundance (CWM) of 82%, followed by medium‐sized (200–600 μm, 11%), and small‐sized (< 200 μm, 5%) organisms. The most common feeding types were tactile raptorial (Cyclopoida, Harpacticoida, and *Leptodora*, 78%), D‐filtration (Daphniidae, 13%), and stationary suspension (Calanoida, 2%). Rotifers with microphagous and raptorial feeding types, along with cladocerans with B‐, C‐, and S‐filtration types, made up less than 2% of the total biomass. Medium‐sized organisms (200–600 μm) were positively influenced by eutrophication until hypereutrophic levels, then declined at TP levels above 300 μg L^−1^. Suspension feeders and D‐filtration feeders were negatively impacted by eutrophication, showing significant decreases in their CWM values as TP increased. Tactile raptorial feeders thrived with eutrophication until TP reached 300 μg L^−1^. Regarding trophic groups, herbivores and omnivores had median CWMs of 22% and 56%, respectively, whereas carnivores and detritivores contributed less than 2%. Eutrophication negatively affected herbivores and positively impacted omnivores (Figure S6; Table S1).

**FIGURE 4:**
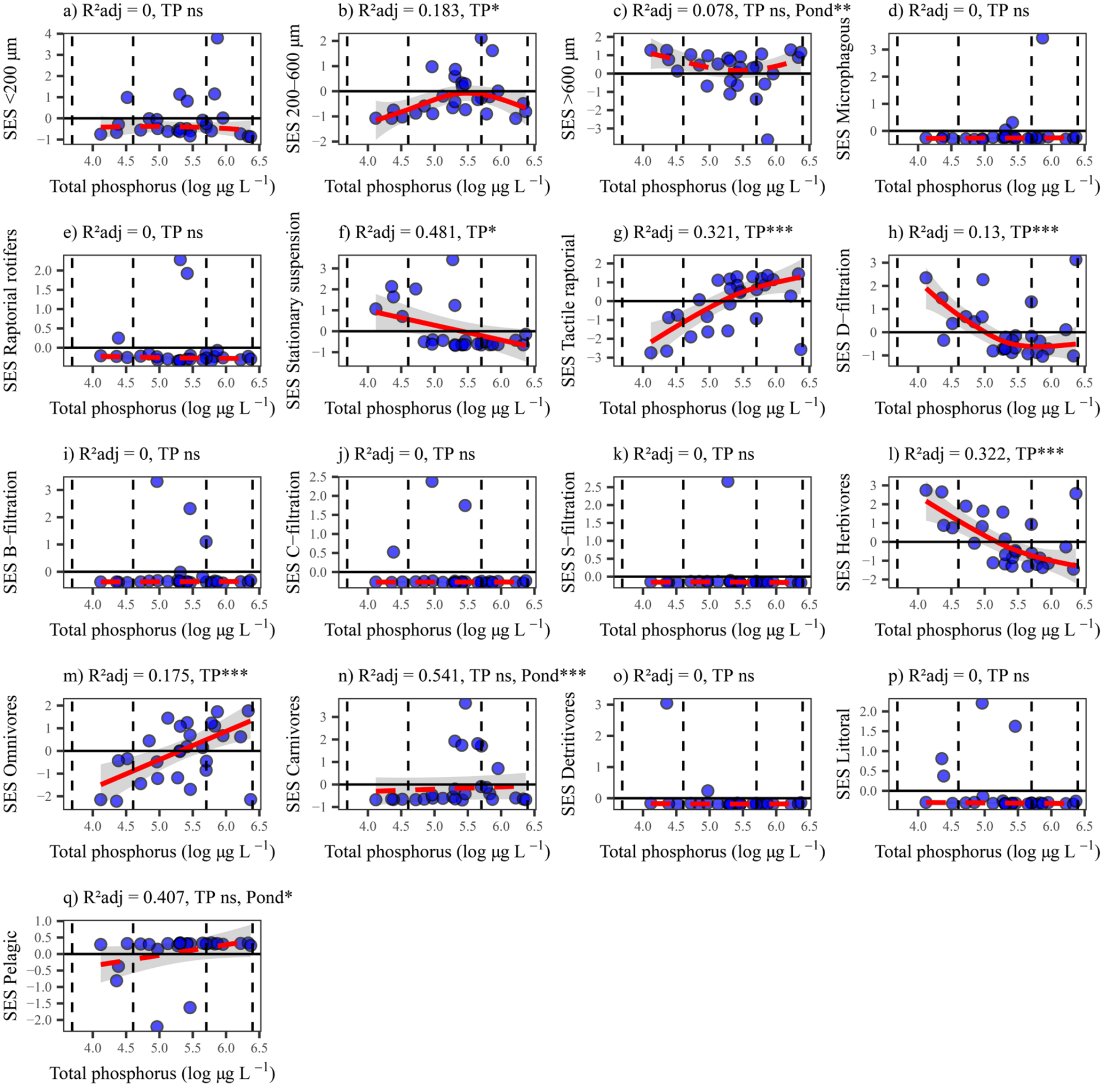
Eutrophication causes trait convergence with contrasting effects on opposing traits. Changes in standardised effect sizes (SES) for community‐weighted means (CWM) of 17 zooplankton functional trait categories along the eutrophication gradient (log‐transformed total phosphorus, TP). Size classes: < 200 µm (a), 200–600 µm (b), and > 600 µm (c); feeding types: microphagous (d), raptorial rotifers (e), stationary suspension (f), tactile‐raptorial (g), D‐ (h), B‐ (i), C‐ (j), and S‐ (k) filtration types; trophic groups: herbivorous (l), omnivorous (m), carnivorous (n), and detritivorous (o); and habitat: littoral (p) and pelagic (q). Values below zero represent trait convergence driven by habitat filtering, and values above zero indicate trait divergence driven by limiting similarity (competition). Models were fitted using generalised additive mixed models (GAMM), accounting for spatial and temporal autocorrelation by adding pond location and sampling time (month) as random factors. Solid red lines represent the significant effects of TP. Vertical dashed lines separate the TP gradient into eutrophic (40–100 μg L^−1^), hypereutrophic (100–300 μg L^−1^), and highly hypereutrophic (> 300 μg L^−1^) levels. ns non‐significant, *p < 0.05, **p < 0.01, ***p < 0.001.

Most functional traits were generally driven by trait convergence (SES < 0 in the entire data; e.g., microphagous, raptorial rotifers, B‐, C‐, and S‐filtration feeders, detritivores, and littoral species), compared to trait divergence (SES > 0; e.g., pelagic species). The SES for the size class 200–600 μm significantly increased with eutrophication until hypereutrophic levels, then declined. The SES for organisms > 600 μm shifted from trait divergence to random at hypereutrophic levels. The SES for stationary suspension, D‐filtration feeders, and herbivores significantly decreased with eutrophication, whereas the SES for tactile‐raptorial feeders and omnivores was positively affected by TP (moving from trait convergence to random together with carnivores). Stationary suspension and herbivores shifted from trait divergence in eutrophic ponds, to random in hypereutrophic, and further to trait convergence under highly hypereutrophic conditions. Littoral species moved from random to trait convergence, and pelagic from random to trait divergence, under highly hypereutrophic states (Figure 4; Tables S2 and S3).

### 3.4 Impacts of Eutrophication and Traits on Zooplankton Diversity

**FIGURE 5:**
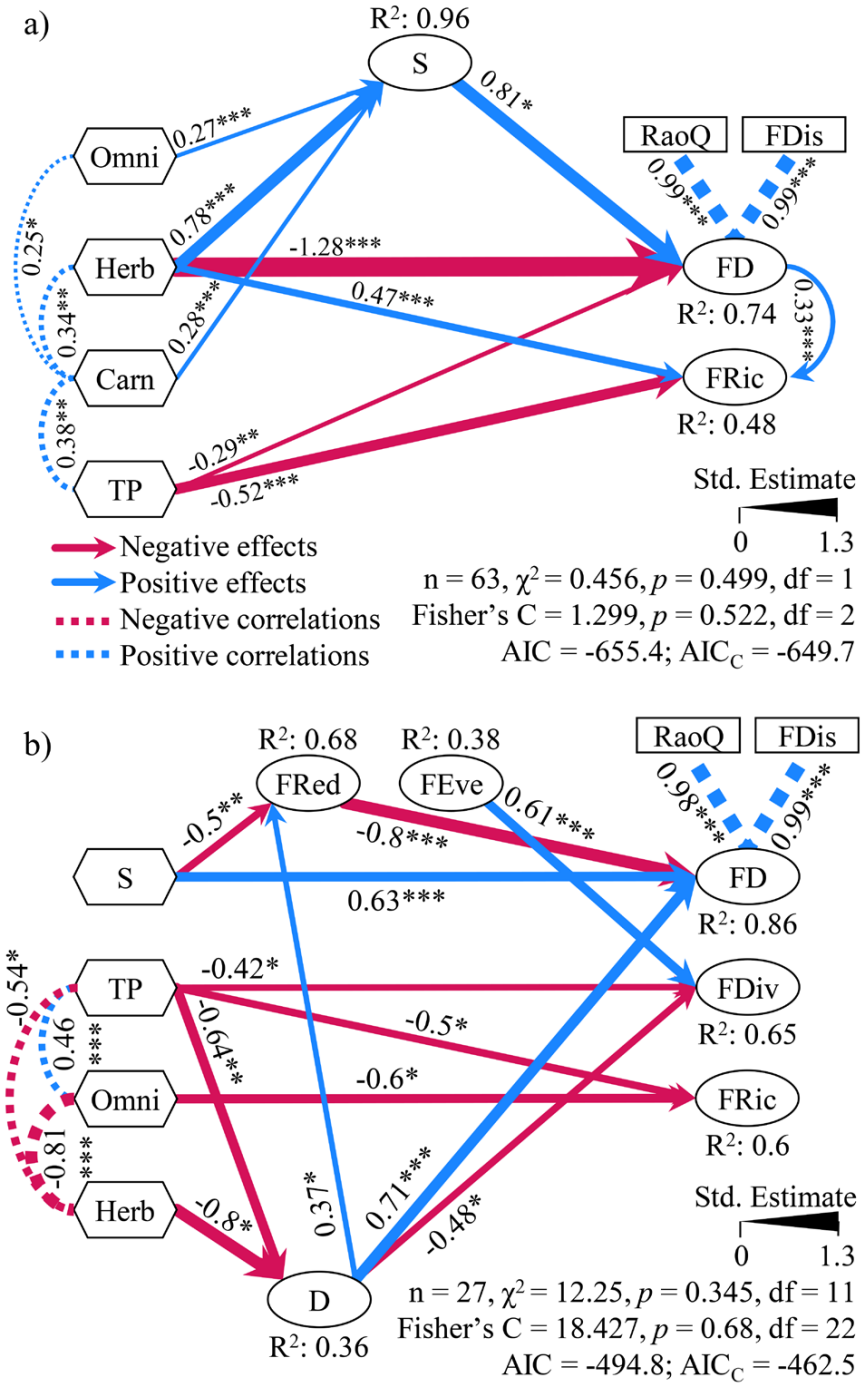
Functional diversity and assembly rules of zooplankton are driven by complex interactions between eutrophication and traits. Piecewise structural equation models (pSEM) identifying direct and indirect effects of eutrophication (log‐transformed total phosphorus, TP), relevant functional traits (Omni: Omnivores; Herb: Herbivores; Carn: Carnivores), and taxonomic diversity (*S*: log‐transformed species richness; *D*: Simpson's diversity index) on functional diversity indices (FD: FDis + RaoQ, latent variable; FRic; FEve; FDiv; FRed). (a) pSEM model using incidence data from March to September 2024 (Omni, Herb, and Carn are the species richnesses of those traits in each sample); (b) pSEM model using biomass data from July to September 2024 (Omni and Herb are the community weighted means of those traits in each sample). Only significant paths are shown: *p < 0.05, **p < 0.01, ***p < 0.001.

Complex direct and indirect effects mediated the impacts of eutrophication on FD through interactions with functional traits and taxonomic diversity. The pSEM model on the basis of the incidence data (Fischer's *C* = 1.299, *p* = 0.522, AIC_*C*_ = –649.7; Figure 5a) revealed that total phosphorus negatively impacted FRic and FD (FDis + RaoQ). The richness of herbivores, omnivores, and carnivores contributed positively to overall species richness, which, in turn, boosted FD. Herbivore richness positively influenced FRic and negatively influenced FD. Both functional diversities were positively correlated with each other. TP increased carnivore richness, which, in turn, positively correlated with omnivore and herbivore richness.

The model on the basis of biomass data (Fischer's *C* = 18.427, *p* = 0.68, AIC_*C*_ = –462.5; Figure 5b) displayed more complex interactions. TP had a negative influence on FRic, FDiv, and Simpson's diversity, whereas omnivore CWM hindered FRic, and herbivores negatively impacted Simpson's diversity. Species richness and Simpson's diversity boosted FD, whereas they had opposite effects on FRed (richness influenced negatively and Simpson's diversity positively); FRed, in turn, diminished FD, revealing direct and indirect effects of taxonomic diversity. FEve boosted FDiv. Total phosphorus harmed herbivores but favoured omnivores, and these two were negatively correlated with each other.

Both bootstrap and Monte Carlo tests supported that all but one path estimate had sufficient statistical power for sample size sufficiency and adequate *p*‐value estimation. The path FEve~species richness in the biomass‐based pSEM (estimate = 0.42, *p* = 0.034) did not persist after empirical bootstrap testing (*p* = 0.104) (Figures S6–S9).

## 4 Discussion

**FIGURE 6:**
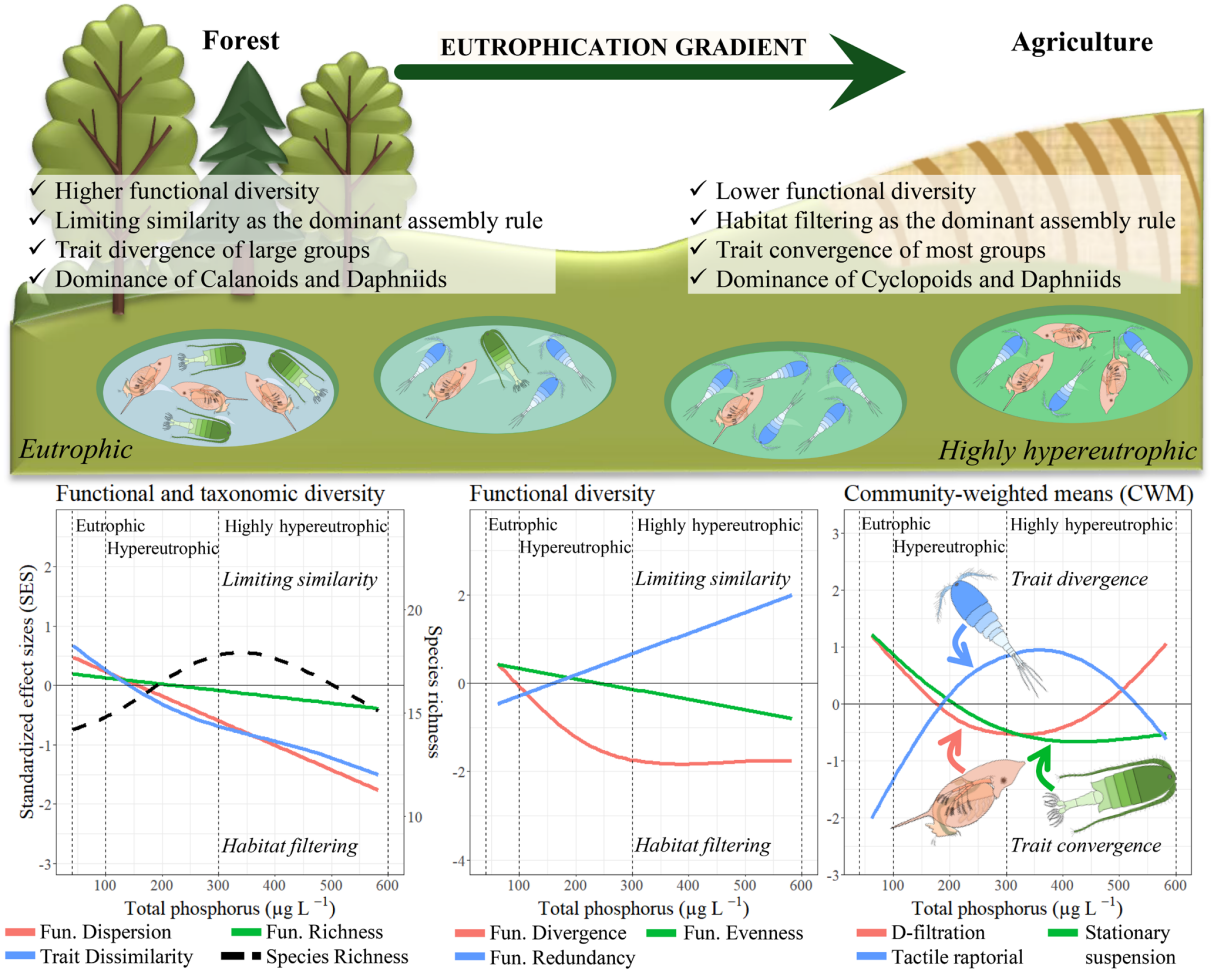
Summary of the main results highlighting the loss of functional diversity, shifts in assembly rules, and alternance in functional guilds due to eutrophication.

This study used taxonomic and trait‐based approaches to evaluate how eutrophication influences zooplankton community assembly in fishponds. We found that extreme eutrophication led to declines in FD and shifts in assembly rules from limiting similarity to habitat filtering, while maintaining species richness stable. Trait convergence across most functional trait categories suggests strong habitat filtering: only species with traits adapted to high nutrient availability are retained, reducing overall FD. Alternatively, the divergence among tactile‐raptorial feeders and omnivores may indicate niche differentiation and competition within those functional guilds. Robust relationships between functional traits, diversity, and TP levels suggest strong links between environmental stressors and zooplankton assembly mechanisms (Figure 6).

The observed reduced Simpson's diversity suggests the dominance of species or functional groups with high biomass production in highly eutrophic environments. The taxonomic and FD of zooplankton were shown to exhibit contrasting responses to eutrophication, with some studies reporting declines (Jeppesen et al. 2000; Moody and Wilkinson 2019; Amorim et al. 2025) or even increases (Vogt et al. 2013). These variations highlight the importance of considering community responses across different eutrophication levels and scales. Studies in temperate lakes have shown that declines in zooplankton taxonomic diversity are often accompanied by increases in biomass under hypereutrophic conditions, with small cladocerans and cyclopoid copepods dominating (Jeppesen et al. 2000).

The response of taxonomic diversity to eutrophication varies with scale and diversity indices used. Although all the study ponds were hypereutrophic, sharp declines in Simpson's diversity occurred at highly hypereutrophic conditions, indicating an imbalance in species' relative biomasses. Species richness, however, exhibited weak and non‐significant variations. Research on large lakes shows a unimodal pattern in species richness, with greater diversity at moderate productivity levels, but being impaired under extreme conditions (Dodson et al. 2000). Shallow lakes and ponds are inherently more productive and have variable richness responses on the basis of geographic scale, taxa, and productivity level, whereas eutrophic and hypereutrophic fishponds show weak species richness declines (Rosset et al. 2014). Fishponds, with lower zooplankton diversity than fishless ponds, still include unique and rare species, indicating the greatest biodiversity potential of small water bodies even under eutrophication (Kuczyńska‐Kippen and Pronin 2018). This supports the exceptional conservation value of small ponds as sources for aquatic biodiversity. Nonetheless, small shifts in taxonomic diversity may hide significant reductions in FD, as species richness alone might not be a proper surrogate for biodiversity changes, requiring the evaluation of multiple facets.

Functional diversity is widely regarded as a key predictor of ecosystem stability and functioning (Van Der Plas 2019; Özgencil et al. 2025). In our study, all FD indices (FRic, FEve, FDiv, FDis, and RaoQ) declined with increasing eutrophication, indicating losses of ecosystem functions in hypereutrophic fishponds. Those metrics are known to strongly correlate with assembly rules: high FD indicates limiting similarity, and low diversity indicates habitat filtering (Mouchet et al. 2010). FRed remained consistently high across all eutrophication levels, but SES for FRed consistently increased under highly hypereutrophic conditions. This pattern is due to the exclusion of traits sensitive to higher eutrophication, leaving unexplored niches for tolerant species belonging to a few traits (redundancy) (Mouchet et al. 2010). Similar patterns have been reported in other freshwater communities, including benthic cyanobacteria (Silva et al. 2025), submerged macrophytes (Cheng et al. 2023), and fish (Feng et al. 2023). For zooplankton, FD indices declined with eutrophication in agricultural and urban shallow ponds (Duré et al. 2021; Fernández‐Aláez et al. 2025).

The consistently high FRed suggests that despite declines in diversity, all ponds maintained high trait redundancies, with the remaining species performing similar ecosystem functions. Functional homogenization, driven by dominant generalist species, prevented the establishment of rare specialists, as observed elsewhere (Lengyel et al. 2023). Although some ecosystems, like coral reefs (Mouillot et al. 2014), exhibit high biodiversity, taxonomic diversity does not always correlate with functional heterogeneity. High species richness may buffer biodiversity loss, but functional over‐redundancy may threaten some underrepresented functions under disturbances (Mouillot et al. 2014). Nevertheless, redundant groups can be more resilient to species loss, as the remaining species compensate for extinct ones (Biggs et al. 2020). In less eutrophic environments, lower trait redundancy makes the ecosystem vulnerable to species loss, with negative consequences for ecosystem stability (Silva et al. 2025).

Functional diversity indices are valuable tools for understanding community assembly patterns (Mouchet et al. 2010). Our results reveal a shift from limiting similarity under eutrophic states (e.g., FRic, FDis, and RaoQ on the basis of incidence) to random and then to niche filtering (e.g., FDiv, FDis, and RaoQ on the basis of biomass) in hypereutrophic and highly hypereutrophic conditions. This implies that eutrophication reduces FD, lowers interspecific competition, and favours groups adapted to high TP levels (e.g., cyclopoid copepods). Notably, only Vogt et al. (2013) studied the assembly rules of zooplankton to date, showing patterns dominated by randomness (> 85% of lakes with TP < 100 μg L^−1^) and rare habitat filtering.

Community shifts along the eutrophication gradient reflected succession patterns in trait categories. Large‐bodied zooplankton (> 600 μm), herbivores, stationary suspension feeders, and D‐filtration feeders are seemingly less tolerant at hypereutrophic levels in fishponds, perhaps because of reduced habitat quality or altered food quality. In contrast, the opposite traits, such as medium‐sized taxa (200–600 μm), omnivorous feeders, and tactile‐raptorial feeders, positively responded to increasing total phosphorus at eutrophic and hypereutrophic levels, potentially taking advantage of increased resources, but declined at highly hypereutrophic conditions (> 300 μg L^−1^ TP), suggesting threshold responses. This pattern has been noted elsewhere, with calanoids (stationary suspension herbivores) declining and cyclopoids (tactile‐raptorial omnivores) increasing with eutrophication (Sommer and Stibor 2002). The slow growth and reproduction rates of calanoids make their larval stages vulnerable to grazing by cyclopoids (Adrian 1997). Daphniids, with faster metabolic and reproductive rates (Sommer and Stibor 2002), rapidly evolve and grow faster under hypereutrophic conditions (Frisch et al. 2014). Those patterns were accompanied by reduced FD at extreme nutrient enrichment, favouring functionally redundant species and homogenising zooplankton communities in the studied fishponds.

Trait convergence governed most traits, whereas divergence was limited to large taxa at less eutrophic levels (e.g., organisms > 600 μm, stationary suspension feeders, and herbivores). This suggests that severe eutrophication promotes a few traits best suited to the conditions found in such fishponds (trait convergence and habitat filtering). Historically, ecologists argued that closely related species are more susceptible to competition, limiting coexistence and facilitating colonisation by distantly related taxa (i.e., trait divergence and limiting similarity) (Grime 2006). Recent findings have indicated that this is rather the exception than the rule, with the need to identify relevant traits capable of distinguishing niche‐ or competition‐driven patterns (Mayfield and Levine 2010). As confirmed in our study, trait convergence is more common and influenced by trait similarity across different habitat gradients (Winemiller et al. 2015).

Eutrophication influenced FD by complex direct and indirect effects that involve interactions between traits and taxonomic diversity. Those complex interactions are proven to have direct influences on ecosystem functioning and stability (Sperandii et al. 2025). The pSEM revealed that although it boosted the abundance of omnivores, eutrophication negatively impacted herbivore abundance, taxonomic, and FD indices. Trophic groups indirectly influenced FD through subsequent decreases in taxonomic diversity (Simpson's index) upon dominance. The dominance of omnivores, one of the groups most influenced by eutrophication, led to declines in FRic, as they were among the few with traits that tolerated highly eutrophic conditions (redundancy). Despite being positively influenced by taxonomic diversity, FD was hindered by the loss of unique functions. When the habitat selects species with similar traits, habitat filtering assembly rules result in lower‐than‐expected FD, thus enhancing the functional redundancy of the remaining traits (de Bello et al. 2009). These results suggest that maintaining larger organisms (e.g., Calanoid copepods and Daphniids) benefits FD by occupying empty niches.

Our study integrates null models with pSEM to uncover the ecological mechanisms influencing zooplankton community assembly. Null models allowed us to rigorously test assumptions against random community assembly processes, providing deeper insights into FD patterns and the distinct roles of habitat filtering versus limiting similarity. Given the linear dependency of SES derived from null models on observed FD and species pools (de Bello 2012), our findings must be applied with caution in similar small and anthropogenically disturbed habitats with similar zooplankton compositions, such as shallow lakes and ponds. pSEM enabled us to disentangle the complex relationships among eutrophication, traits, taxonomic, and functional diversity, fostering robust causal inferences while reducing the risk of spurious correlations. Although the need for higher seasonal resolution has long been recognised as a critical gap in assembly rule studies (Weiher and Keddy 1995; Shinohara et al. 2023) and explored herein, long‐term patterns of community assembly in freshwater communities remain understudied (e.g., Kuczynski and Grenouillet 2018). Among the limitations of our study is its narrow spatial resolution and the heterogeneity of the habitats studied. Since our study was confined to eutrophic and hypereutrophic systems, with identical results to other anthropogenically impacted ponds (e.g., Fernandez‐Fournier and Avilés 2018; Kuczyńska‐Kippen and Pronin 2018), the lower end of the eutrophication gradient was not explored. Thus, further studies at larger temporal and spatial scales are necessary, as community assembly rules and responses in oligotrophic and mesotrophic environments may differ from our findings, and metacommunities might evolve over time.

## 5 Conclusions

In conclusion, this research recorded sharp declines of zooplankton FD under severe eutrophication in fishponds. Community assembly rules were largely regulated by trait convergence and habitat filtering, confirming our hypothesis. The findings present a new perspective on the stress‐dominance hypothesis, originally developed for vegetation, which posits that poor nutrient and stressful conditions impair communities by inducing trait convergence through habitat filtering (Coyle et al. 2014). By applying this hypothesis to freshwater zooplankton, we showed that habitat filtering and trait convergence continued to rule zooplankton assemblages under extreme conditions, though from a reverse perspective, linking excessive nutrient supply, rather than limitation, to environmental stress. The highly hypereutrophic conditions, the extreme end of the productivity gradient in aquatic ecosystems, strongly harmed some functional groups but favoured others (e.g., tactile‐raptorial feeders and omnivores).

Our findings indicate that extensively managed hypereutrophic fishponds can maintain stable and high zooplankton richness, especially when total phosphorus remains below 300 μg L^−1^. However, this could cover up the detrimental effects of eutrophication on other aspects of biodiversity, for example, FD. The functional homogenization triggered by eutrophication underscores the need for multidisciplinary approaches to assess species responses to environmental stressors beyond species inventories. To ensure FD and stability in fishponds, pond managers should implement measures to promote sustainable fish production and biodiversity conservation, for example, by reducing nutrient inputs from agriculture and fish feeds.

## Author Contributions

C.A.A. contributed to conceptualisation, methodology, validation, data curation, visualisation, formal analysis, and wrote the first draft of the manuscript. M.J.K. contributed to methodology, resource acquisition, and project administration. C.A.A. and M.J.K. contributed substantially to revisions and approved the submission.

## Funding

This work was supported by Amt der NÖ Landesregierung (K3‐F‐913/004‐2023). Open access funding was provided by WasserCluster Lunz—Biologische Station GmbH.

## Conflicts of Interest

The authors declare no conflicts of interest.

## Supporting information


**Data S1:** ele70289‐sup‐0001‐FigS1‐S9‐TableS1‐S3.pdf.

## Data Availability

The associated data and R codes are openly available in Figshare at https://doi.org/10.6084/m9.figshare.29400842 (Amorim and Kainz 2025).

## References

[ele70289-bib-0001] Adrian, R. 1997. “Calanoid‐Cyclopoid Interactions: Evidence From an 11‐Year Field Study in a Eutrophic Lake.” Freshwater Biology 38: 315–325. 10.1046/J.1365-2427.1997.00215.X.

[ele70289-bib-0002] Amorim, C. A. , E. Jeppesen , and A. N. Moura . 2025. “How Do Additions of Submerged Macrophytes, Large‐Bodied Cladocerans and Nutrients Impact Tropical Plankton Communities? A Mesocosm Experiment.” Hydrobiologia 852: 489–501. 10.1007/s10750-024-05646-8.

[ele70289-bib-0003] Amorim, C. A. , and M. J. Kainz . 2025. “Data From: Shifts in Assembly Rules and Loss of Zooplankton Functional Diversity Across Hypereutrophic Fishponds.” *Figshare* . 10.6084/m9.figshare.29400842. PMC12698031 41379767

[ele70289-bib-0004] Amorim, C. A. , and A. d. N. Moura . 2021. “Ecological Impacts of Freshwater Algal Blooms on Water Quality, Plankton Biodiversity, Structure, and Ecosystem Functioning.” Science of the Total Environment 758: 143605. 10.1016/j.scitotenv.2020.143605. 33248793

[ele70289-bib-0005] Amorim, C. A. , and A. d. N. Moura . 2022. “Habitat Templates of Phytoplankton Functional Groups in Tropical Reservoirs as a Tool to Understand Environmental Changes.” Hydrobiologia 849: 1095–1113. 10.1007/s10750-021-04750-3.

[ele70289-bib-0006] Barnett, A. J. , K. Finlay , and B. E. Beisner . 2007. “Functional Diversity of Crustacean Zooplankton Communities: Towards a Trait‐Based Classification.” Freshwater Biology 52: 796–813. 10.1111/j.1365-2427.2007.01733.x.

[ele70289-bib-0007] Biggs, C. R. , L. A. Yeager , D. G. Bolser , et al. 2020. “Does Functional Redundancy Affect Ecological Stability and Resilience? A Review and Meta‐Analysis.” Ecosphere 11: e03184. 10.1002/ecs2.3184.

[ele70289-bib-0008] Borics, G. , V. B‐Béres , I. Bácsi , et al. 2020. “Trait Convergence and Trait Divergence in Lake Phytoplankton Reflect Community Assembly Rules.” Scientific Reports 10: 1–11. 10.1038/s41598-020-76645-7. 33177646 PMC7658209

[ele70289-bib-0009] Cadotte, M. W. , R. Dinnage , and D. Tilman . 2012. “Phylogenetic Diversity Promotes Ecosystem Stability.” Ecology 93: S223–S233. 10.1890/11-0426.1.

[ele70289-bib-0010] Cardinale, B. J. , J. E. Duffy , A. Gonzalez , et al. 2012. “Biodiversity Loss and Its Impact on Humanity.” Nature 486, no. 7401: 59–67. 10.1038/nature11148. 22678280

[ele70289-bib-0011] Céréghino, R. , D. Boix , H.‐M. Cauchie , K. Martens , B. Oertli , and R. Céréghino . 2014. “The Ecological Role of Ponds in a Changing World.” Hydrobiologia 723: 1–6. 10.1007/s10750-013-1719-y.

[ele70289-bib-0012] Cheng, C. , J. Chen , H. Su , et al. 2023. “Eutrophication Decreases Ecological Resilience by Reducing Species Diversity and Altering Functional Traits of Submerged Macrophytes.” Global Change Biology 29: 5000–5013. 10.1111/gcb.16872. 37428468

[ele70289-bib-0013] Coyle, J. R. , F. W. Halliday , B. E. Lopez , K. A. Palmquist , P. A. Wilfahrt , and A. H. Hurlbert . 2014. “Using Trait and Phylogenetic Diversity to Evaluate the Generality of the Stress‐Dominance Hypothesis in Eastern North American Tree Communities.” Ecography 37: 814–826. 10.1111/ecog.00473.

[ele70289-bib-0014] Craven, D. , N. Eisenhauer , W. D. Pearse , et al. 2018. “Multiple Facets of Biodiversity Drive the Diversity–Stability Relationship.” Nature Ecology & Evolution 2, no. 10: 1579–1587. 10.1038/s41559-018-0647-7. 30150740

[ele70289-bib-0015] de Bello, F. 2012. “The Quest for Trait Convergence and Divergence in Community Assembly: Are Null‐Models the Magic Wand?” Global Ecology and Biogeography 21: 312–317. 10.1111/J.1466-8238.2011.00682.X.

[ele70289-bib-0016] de Bello, F. , W. Thuiller , J. Leps , et al. 2009. “Partitioning of Functional Diversity Reveals the Scale and Extent of Trait Convergence and Divergence.” Journal of Vegetation Science 20: 475–486. 10.1111/J.1654-1103.2009.01042.X.

[ele70289-bib-0017] Dodson, S. I. , S. E. Arnott , and K. L. Cottingham . 2000. “The Relationship in Lake Communities Between Primary Productivity and Species Richness.” Ecology 81: 2662–2679. 10.1890/0012-9658(2000)081[2662:TRILCB]2.0.CO;2.

[ele70289-bib-0018] Dudgeon, D. 2019. “Multiple Threats Imperil Freshwater Biodiversity in the Anthropocene.” Current Biology 29: R960–R967. 10.1016/j.cub.2019.08.002. 31593677

[ele70289-bib-0019] Dumont, H. J. , I. Van de Velde , and S. Dumont . 1975. “The Dry Weight Estimate of Biomass in a Selection of Cladocera, Copepoda and Rotifera From the Plankton, Periphyton and Benthos of Continental Waters.” Oecologia 19: 75–97. 10.1007/BF00377592. 28308833

[ele70289-bib-0020] Duré, G. A. V. , N. R. Simões , L. D. S. M. Braghin , and S. M. M. S. Ribeiro . 2021. “Effect of Eutrophication on the Functional Diversity of Zooplankton in Shallow Ponds in Northeast Brazil.” Journal of Plankton Research 43: 894–907. 10.1093/PLANKT/FBAB064.

[ele70289-bib-0021] Ejsmont‐Karabin, J. 1998. “Empirical Equations for Biomass Calculation of Planktonic Rotifers.” Polskie Archiwum Hydrobiologii 4: 513–522.

[ele70289-bib-0022] Feng, K. , W. Deng , Y. Zhang , et al. 2023. “Eutrophication Induces Functional Homogenization and Traits Filtering in Chinese Lacustrine Fish Communities.” Science of the Total Environment 857: 159651. 10.1016/J.SCITOTENV.2022.159651. 36280085

[ele70289-bib-0023] Fernández‐Aláez, C. , S. Manzanal , M. Fernández‐Aláez , and J. García‐Girón . 2025. “Deciphering the Patterns and Correlates of Zooplankton Functional Diversity in Mountain and Lowland Ponds.” Freshwater Biology 70: e14378. 10.1111/FWB.14378.

[ele70289-bib-0024] Fernandez‐Fournier, P. , and L. Avilés . 2018. “Environmental Filtering and Dispersal as Drivers of Metacommunity Composition: Complex Spider Webs as Habitat Patches: Complex.” Ecosphere 9: e02101. 10.1002/ecs2.2101.

[ele70289-bib-0025] Frisch, D. , P. K. Morton , P. R. Chowdhury , et al. 2014. “A Millennial‐Scale Chronicle of Evolutionary Responses to Cultural Eutrophication in Daphnia.” Ecology Letters 17: 360–368. 10.1111/ELE.12237. 24400978

[ele70289-bib-0026] Gotelli, N. J. 2000. “Null Model Analysis of Species Co‐Occurrence Patterns.” Ecology 81: 2606–2621. 10.1890/0012-9658(2000)081[2606:NMAOSC]2.0.CO;2.

[ele70289-bib-0027] Götzenberger, L. , F. de Bello , K. A. Bråthen , et al. 2012. “Ecological Assembly Rules in Plant Communities—Approaches, Patterns and Prospects.” Biological Reviews 87: 111–127. 10.1111/J.1469-185X.2011.00187.X. 21692965

[ele70289-bib-0028] Grime, J. P. 2006. “Trait Convergence and Trait Divergence in Herbaceous Plant Communities: Mechanisms and Consequences.” Journal of Vegetation Science 17: 255–260. 10.1111/J.1654-1103.2006.TB02444.X.

[ele70289-bib-0029] Hansen, H. P. , and F. Koroleff . 1999. “Determination of Nutrients.” In Methods of Seawater Analysis, edited by K. Grasshoff , K. Kremling , and M. Ehrhardt , 159–228. Wiley‐VCH. 10.1002/9783527613984.CH10.

[ele70289-bib-0030] Hutchinson, G. E. 1961. “The Paradox of the Plankton.” American Naturalist 95: 137–145. 10.1086/282171.

[ele70289-bib-0031] Jeppesen, E. , H. He , M. Søndergaard , et al. 2025. “Experimental Evidence of the Role of Nitrogen for Eutrophication in Shallow Lakes: A Long‐Term Climate Effect Mesocosm Study.” Innovation 6: 100756. 10.1016/J.XINN.2024.100756. 40470332 PMC12131006

[ele70289-bib-0032] Jeppesen, E. , J. P. Jensen , M. Sondergaard , T. Lauridsen , and F. Landkildehus . 2000. “Trophic Structure, Species Richness and Biodiversity in Danish Lakes: Changes Along a Phosphorus Gradient.” Freshwater Biology 45: 201–218. 10.1046/j.1365-2427.2000.00675.x.

[ele70289-bib-0033] Klais, R. , V. Norros , S. Lehtinen , T. Tamminen , and K. Olli . 2017. “Community Assembly and Drivers of Phytoplankton Functional Structure.” Functional Ecology 31: 760–767. 10.1111/1365-2435.12784.

[ele70289-bib-0034] Kuczyńska‐Kippen, N. , and M. Pronin . 2018. “Diversity and Zooplankton Species Associated With Certain Hydroperiods and Fish State in Field Ponds.” Ecological Indicators 90: 171–178. 10.1016/J.ECOLIND.2018.03.016.

[ele70289-bib-0035] Kuczynski, L. , and G. Grenouillet . 2018. “Community Disassembly Under Global Change: Evidence in Favor of the Stress‐Dominance Hypothesis.” Global Change Biology 24: 4417–4427. 10.1111/GCB.14320. 29788536

[ele70289-bib-0036] Laliberte, E. , and P. Legendre . 2010. “A Distance‐Based Framework for Measuring Functional Diversity From Multiple Traits.” Ecology 91: 299–305. 10.1890/08-2244.1. 20380219

[ele70289-bib-0037] Lefcheck, J. S. 2016. “piecewiseSEM: Piecewise Structural Equation Modelling in r for Ecology, Evolution, and Systematics.” Methods in Ecology and Evolution 7: 573–579. 10.1111/2041-210X.12512.

[ele70289-bib-0038] Lengyel, E. , C. Stenger‐Kovács , G. Boros , T. J. K. Al‐Imari , Z. Novák , and G. Bernát . 2023. “Anticipated Impacts of Climate Change on the Structure and Function of Phytobenthos in Freshwater Lakes.” Environmental Research 238: 117283. 10.1016/j.envres.2023.117283. 37783333

[ele70289-bib-0039] Litchman, E. , M. D. Ohman , and T. Kiørboe . 2013. “Trait‐Based Approaches to Zooplankton Communities.” Journal of Plankton Research 35: 473–484. 10.1093/PLANKT/FBT019.

[ele70289-bib-0040] Mack, H. R. , J. D. Conroy , K. A. Blocksom , R. A. Stein , and S. A. Ludsin . 2012. “A Comparative Analysis of Zooplankton Field Collection and Sample Enumeration Methods.” Limnology and Oceanography: Methods 10: 41–53. 10.4319/lom.2012.10.41.

[ele70289-bib-0041] Martini, S. , F. Larras , A. Boyé , et al. 2021. “Functional Trait‐Based Approaches as a Common Framework for Aquatic Ecologists.” Limnology and Oceanography 66: 965–994. 10.1002/LNO.11655.

[ele70289-bib-0042] Mayfield, M. M. , and J. M. Levine . 2010. “Opposing Effects of Competitive Exclusion on the Phylogenetic Structure of Communities.” Ecology Letters 13: 1085–1093. 10.1111/J.1461-0248.2010.01509.X. 20576030

[ele70289-bib-0043] Meyer, M. F. , B. M. Kraemer , C. C. Barbosa , et al. 2025. “Clarifying the Trophic State Concept to Advance Macroscale Freshwater Science and Management.” Ecosphere 16: e70392. 10.1002/ECS2.70392.

[ele70289-bib-0044] Moody, E. K. , and G. M. Wilkinson . 2019. “Functional Shifts in Lake Zooplankton Communities With Hypereutrophication.” Freshwater Biology 64: 608–616. 10.1111/FWB.13246.

[ele70289-bib-0045] Mouchet, M. A. , S. Villéger , N. W. H. Mason , and D. Mouillot . 2010. “Functional Diversity Measures: An Overview of Their Redundancy and Their Ability to Discriminate Community Assembly Rules.” Functional Ecology 24: 867–876. 10.1111/J.1365-2435.2010.01695.X.

[ele70289-bib-0046] Mouillot, D. , S. Villéger , V. Parravicini , et al. 2014. “Functional Over‐Redundancy and High Functional Vulnerability in Global Fish Faunas on Tropical Reefs.” Proceedings of the National Academy of Sciences 111: 13757–13762. 10.1073/pnas.1317625111. PMC4183327 25225388

[ele70289-bib-0047] Nürnberg, G. K. 1996. “Trophic State of Clear and Colored, Soft‐ and Hardwater Lakes With Special Consideration of Nutrients, Anoxia, Phytoplankton and Fish.” Lake and Reservoir Management 12: 432–447. 10.1080/07438149609354283.

[ele70289-bib-0048] Obertegger, U. , and G. Flaim . 2015. “Community Assembly of Rotifers Based on Morphological Traits.” Hydrobiologia 753: 31–45. 10.1007/s10750-015-2191-7.

[ele70289-bib-0049] Özgencil, İ. K. , M. A. Çolak , G. Yılmaz , et al. 2025. “Changes in Diversity of Wetland Birds Across Spatial Scales Following 20 Years of Wetland Degradation: A Case Study From Central Türkiye.” Inland Waters 15: 2488655. 10.1080/20442041.2025.2488655.

[ele70289-bib-0050] Pennekamp, F. , M. Pontarp , A. Tabi , et al. 2018. “Biodiversity Increases and Decreases Ecosystem Stability.” Nature 563, no. 7729: 109–112. 10.1038/s41586-018-0627-8. 30333623

[ele70289-bib-0051] Podani, J. 1999. “Extending Gower's General Coefficient of Similarity to Ordinal Characters.” Taxon 48: 331–340. 10.2307/1224438.

[ele70289-bib-0052] Ricotta, C. , F. de Bello , M. Moretti , M. Caccianiga , B. E. L. Cerabolini , and S. Pavoine . 2016. “Measuring the Functional Redundancy of Biological Communities: A Quantitative Guide.” Methods in Ecology and Evolution 7: 1386–1395. 10.1111/2041-210X.12604.

[ele70289-bib-0053] Rosset, V. , S. Angélibert , F. Arthaud , et al. 2014. “Is Eutrophication Really a Major Impairment for Small Waterbody Biodiversity?” Journal of Applied Ecology 51: 415–425. 10.1111/1365-2664.12201.

[ele70289-bib-0054] Sayer, C. A. , E. Fernando , R. R. Jimenez , et al. 2025. “One‐Quarter of Freshwater Fauna Threatened With Extinction.” Nature 638: 138–145. 10.1038/s41586-024-08375-z. 39779863 PMC11798842

[ele70289-bib-0055] Shinohara, N. , R. Nakadai , Y. Suzuki , and A. Terui . 2023. “Spatiotemporal Dimensions of Community Assembly.” Population Ecology 65: 5–16. 10.1002/1438-390X.12144.

[ele70289-bib-0056] Silva, F. S. , A. N. Moura , and C. A. Amorim . 2025. “Eutrophication Drives Functional and Beta Diversity Loss in Epiphytic Cyanobacteria.” Hydrobiologia 852: 4459–4474. 10.1007/s10750-025-05870-w.

[ele70289-bib-0057] Sinha, E. , A. M. Michalak , and V. Balaji . 2017. “Eutrophication Will Increase During the 21st Century as a Result of Precipitation Changes.” Science 357: 405–408. 10.1126/science.aan2409. 28751610

[ele70289-bib-0058] Smith, V. H. , and D. W. Schindler . 2009. “Eutrophication Science: Where Do We Go From Here?” Trends in Ecology & Evolution 24: 201–207. 10.1016/j.tree.2008.11.009. 19246117

[ele70289-bib-0059] Sommer, U. , and H. Stibor . 2002. “Copepoda – Cladocera – Tunicata: The Role of Three Major Mesozooplankton Groups in Pelagic Food Webs.” Ecological Research 17: 161–174. 10.1046/j.1440-1703.2002.00476.x.

[ele70289-bib-0060] Sperandii, M. G. , M. Bazzichetto , L. Götzenberger , et al. 2025. “Functional Traits Mediate the Effect of Land Use on Drivers of Community Stability Within and Across Trophic Levels.” Science Advances 11: 6445. 10.1126/SCIADV.ADP6445. PMC11759044 39854460

[ele70289-bib-0061] Thulin, M. 2024. Modern Statistics With R. 2n ed. Chapman and Hall/CRC. 10.1201/9781003401339.

[ele70289-bib-0062] Tilman, D. , P. B. Reich , and J. M. H. Knops . 2006. “Biodiversity and Ecosystem Stability in a Decade‐Long Grassland Experiment.” Nature 441: 629–632. 10.1038/nature04742. 16738658

[ele70289-bib-0063] Van Der Plas, F. 2019. “Biodiversity and Ecosystem Functioning in Naturally Assembled Communities.” Biological Reviews 94: 1220–1245. 10.1111/brv.12499. 30724447

[ele70289-bib-0064] Vogt, R. J. , P. R. Peres‐Neto , B. E. Beisner , R. J. Vogt , P. R. Peres‐Neto , and B. E. Beisner . 2013. “Using Functional Traits to Investigate the Determinants of Crustacean Zooplankton Community Structure.” Oikos 122: 1700–1709. 10.1111/J.1600-0706.2013.00039.X.

[ele70289-bib-0065] Weiher, E. , and P. A. Keddy . 1995. “Assembly Rules, Null Models, and Trait Dispersion: New Questions From Old Patterns.” Oikos 74: 159. 10.2307/3545686.

[ele70289-bib-0066] Wezel, A. , B. Oertli , V. Rosset , et al. 2014. “Biodiversity Patterns of Nutrient‐Rich Fish Ponds and Implications for Conservation.” Limnology (Tokyo) 15: 213–223. 10.1007/s10201-013-0419-7.

[ele70289-bib-0067] Winemiller, K. O. , D. B. Fitzgerald , L. M. Bower , and E. R. Pianka . 2015. “Functional Traits, Convergent Evolution, and Periodic Tables of Niches.” Ecology Letters 18: 737–751. 10.1111/ELE.12462. 26096695 PMC4744997

[ele70289-bib-0068] Wood, S. N. 2017. “Generalized Additive Models.” In Generalized Additive Models: An Introduction With R, Second Edition, 2nd ed. Chapman and Hall/CRC. 10.1201/9781315370279.

