# Supplementary material for: Shifts in Assembly Rules and Loss of Zooplankton Functional Diversity Across Hypereutrophic Fishponds
Source: Ecol Lett. 2025 Dec 11;28(12):e70289. doi: 10.1111/ele.70289 (PMC12698031; doi:10.1111/ele.70289)
Supplement: Supplementary file 1 — Data S1: ele70289‐sup‐0001‐FigS1‐S9‐TableS1‐S3.pdf. [file ELE-28-0-s001.pdf]

SUPPORTING INFORMATION

Journal: Ecology Letters

Shifts in assembly rules and loss of zooplankton functional diversity across hypereutrophic fishponds

Running title: Biodiversity loss caused by eutrophication

Cihelio A. Amorim[a,b]* & Martin J. Kainz[a,b]

[a]WasserCluster Lunz – Biologische Station GmbH, Dr. Carl Kupelwieser Promenade 5, 3293, Lunz am See, Austria.
[b]Research Lab for Aquatic Ecosystem Research and Health, University for Continuing Education – Danube University Krems, Dr. Karl Dorrek Straße 30, 3500, Krems an der Donau, Austria

Authors' information:
*CAA – ORCID 0000-0002-7171-7450; Email: alvescihelio@gmail.com, cihelio.amorim@wcl.ac.at (corresponding author)
MJK – ORCID 0000-0002-2388-1504; Email: martin.kainz@donau-uni.ac.at

Keywords: Assembly rules; Competition; Eutrophication; Functional homogenization; Habitat filtering; Limiting similarity; Pond biodiversity; Stress-dominance hypothesis; Threats to biodiversity; Trait convergence.

Data availability

The associated data and R codes are openly available in Figshare at https://doi.org/10.6084/m9.figshare.29400842 (Amorim & Kainz 2025).

Amorim, C.A. & Kainz, M.J. (2025). *Data from: "Shifts in assembly rules and loss of zooplankton functional diversity across hypereutrophic fishponds." Figshare*. Available at: https://doi.org/10.6084/m9.figshare.29400842.

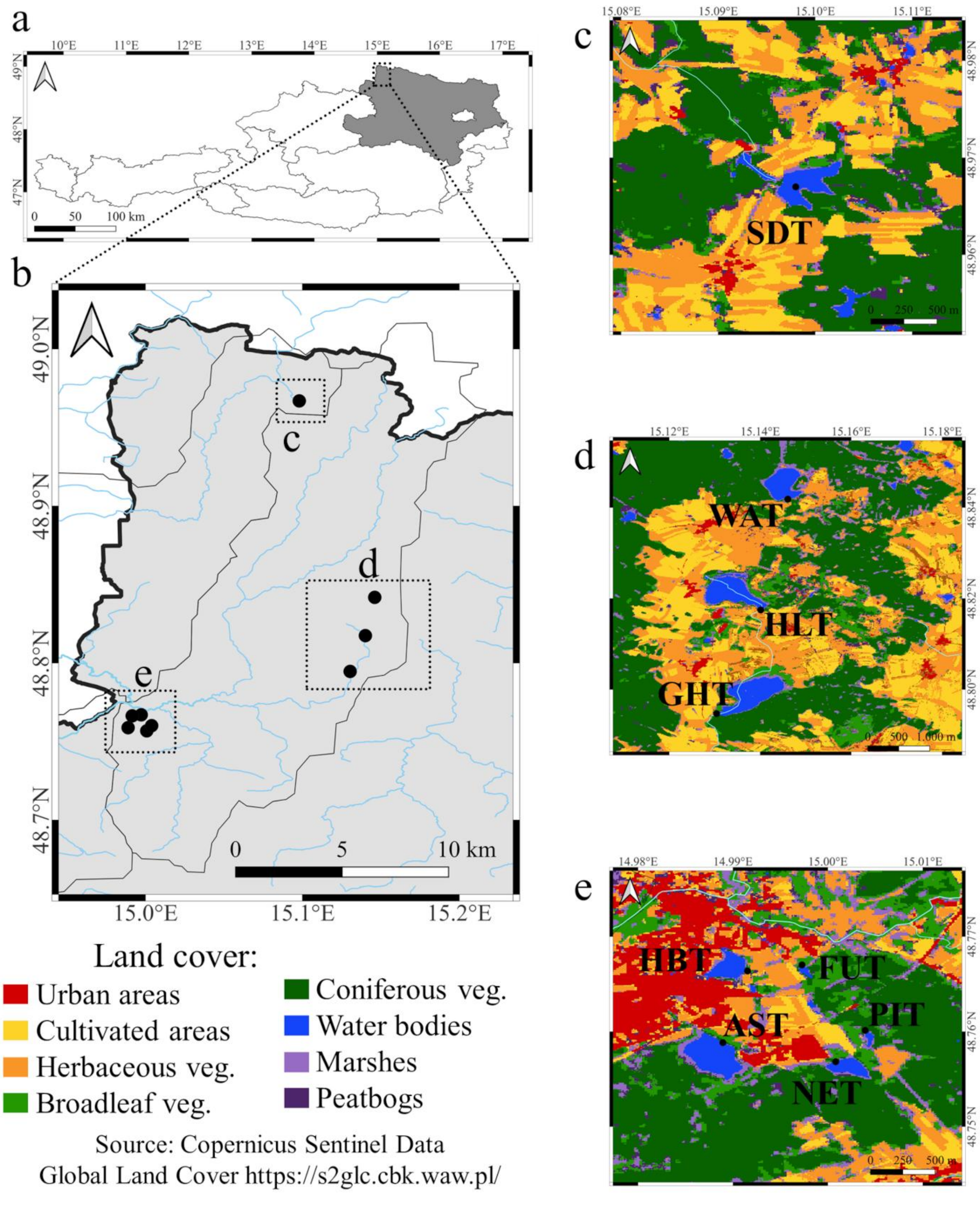

Figure S1 | Geographical location and land use types of the studied fishponds in the Gmünd district (black dots), state of Lower Austria, Austria (a–b). Panels c–e show the land cover types around the ponds. AST: Asang Teich, WAT: Winkelauer Teich, NET: Neu Teich, SDT: Schandachen Teich, FUT: Fuchs Teich, HLT: Haslawer Teich, GHT: Gebharts Teich, PIT: Pilz Teich, HBT: Großer Harabruck Teich.

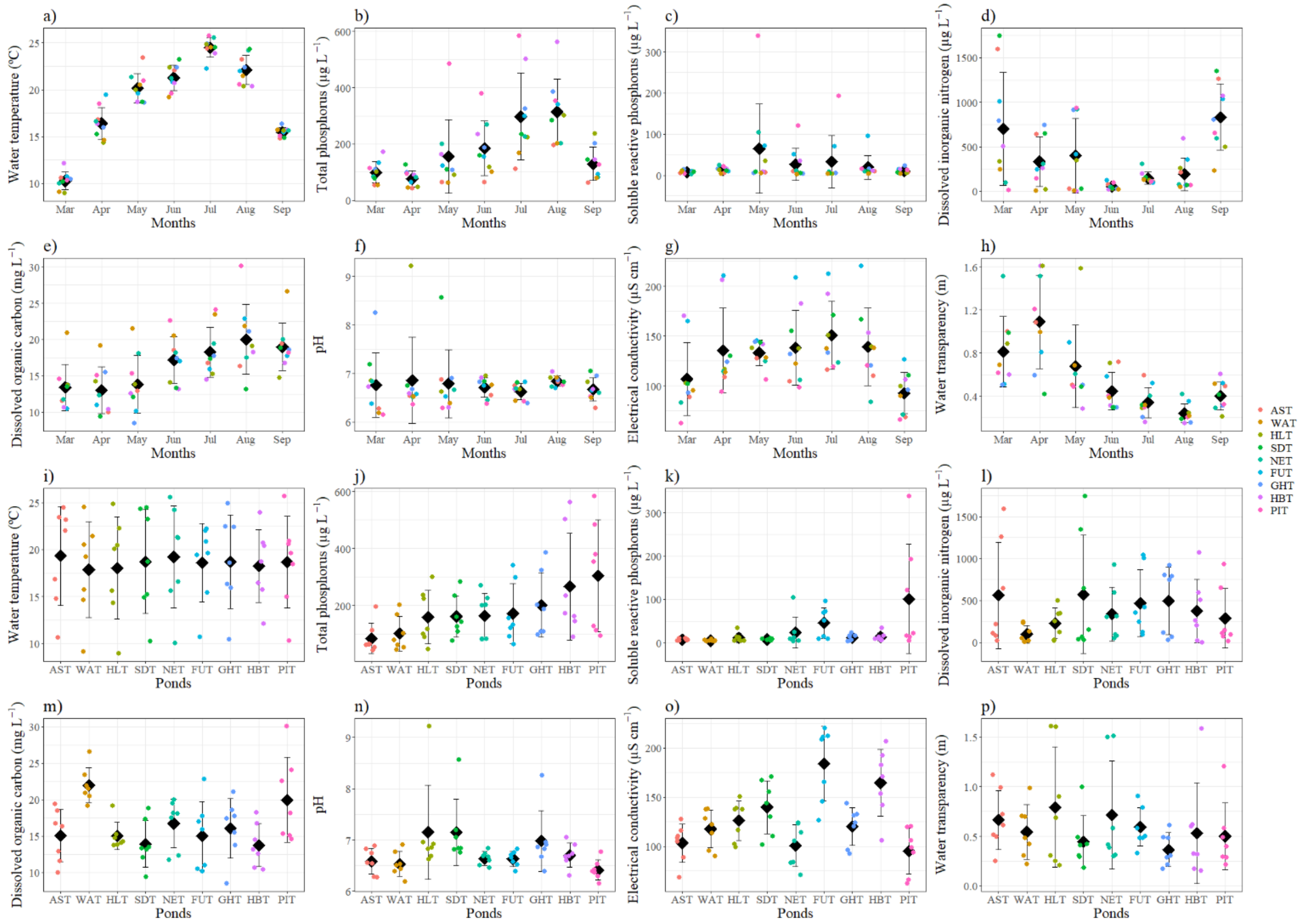

Figure S2 | Water temperature (a, i), concentration of total phosphorus (b, j), soluble reactive phosphorus (c, k), dissolved inorganic nitrogen (d, l), dissolved organic carbon (e, m), pH (f, n), electrical conductivity (g, o), and water transparency (h, p) in the studied fishponds across sampling months (a–h) and ponds (i–p). Ponds are sorted based on the eutrophication gradient. See Figure S1 for abbreviations.

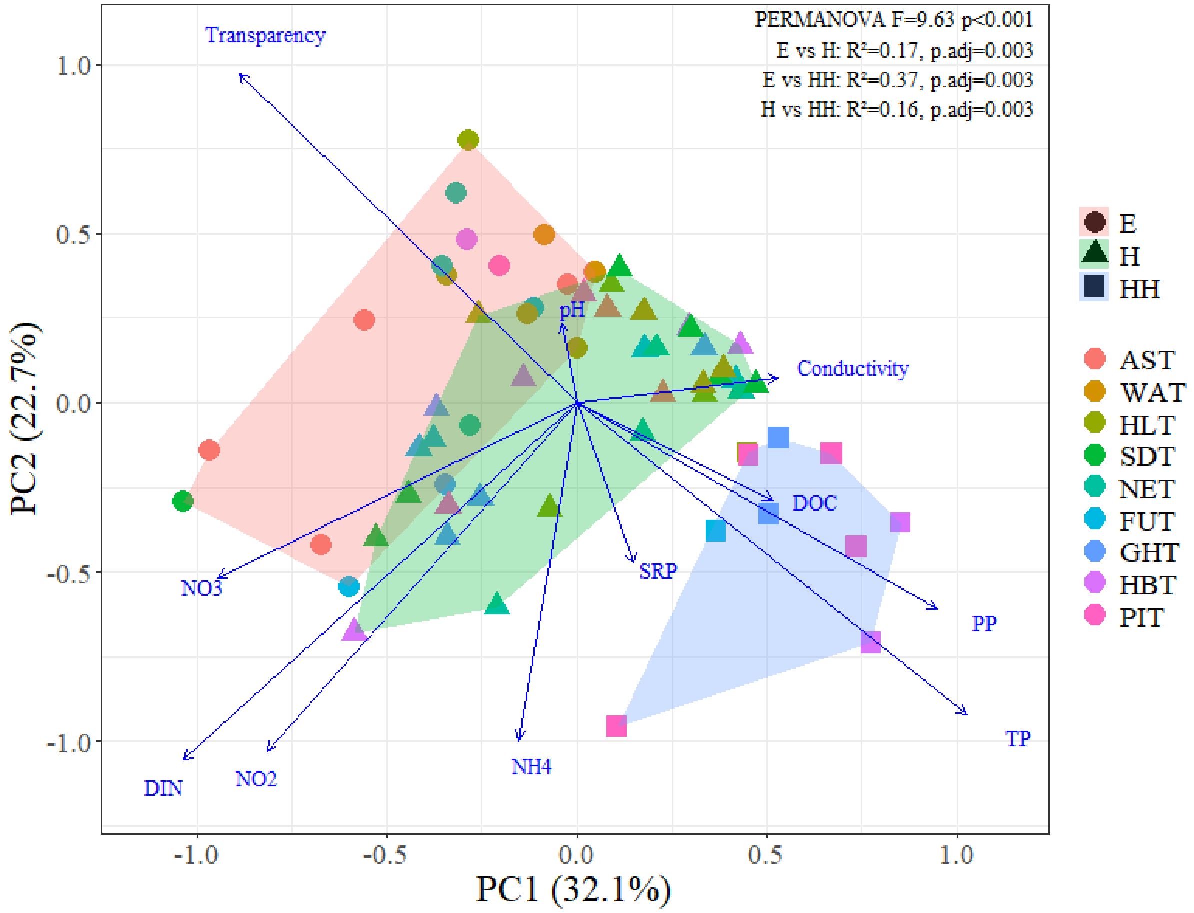

Figure S3 | Principal component analysis (PCA) of physicochemical variables across the studied fishponds. Convex hulls represent trophic state groups (E: Eutrophic; H: Hypereutrophic; HH: Highly hypereutrophic). PERMANOVA comparisons of each pair of trophic states are annotated on the plot. See Figure S1 for pond abbreviations.

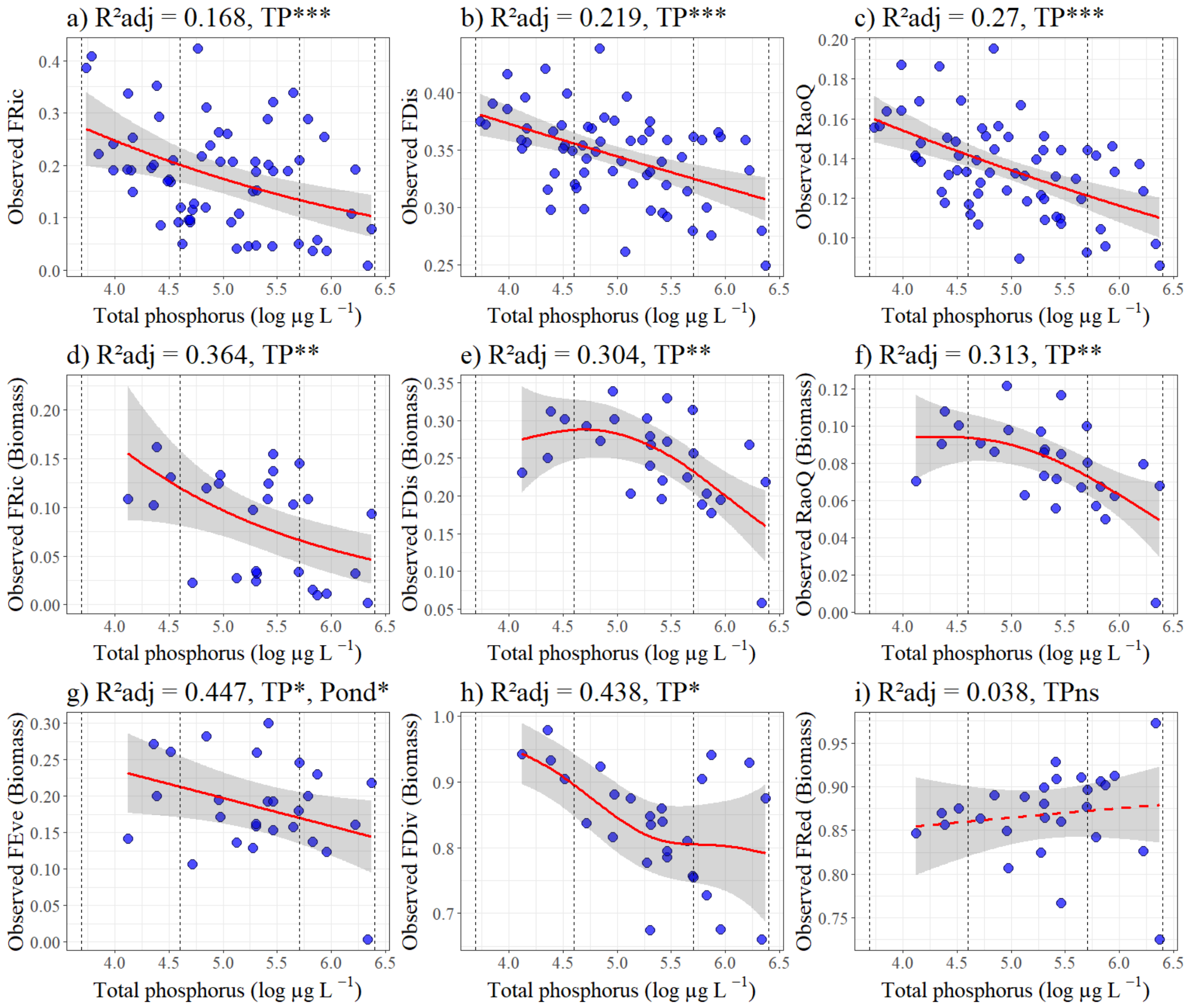

Figure S4 | Observed functional diversity metrics from the incidence data: functional richness (FRic, a), dispersion (FDis, b), and trait dissimilarity (RaoQ, c); and from biomass data: functional richness (FRic, d), dispersion (FDis, e), trait dissimilarity (RaoQ, f), evenness (FEve, g), divergence (FDiv, h), and redundancy (FRed, i) along the eutrophication gradient (log-transformed total phosphorus, TP). Models were fitted using generalized additive mixed models (GAMM), accounting for spatial and temporal autocorrelation by adding pond location and sampling time (month) as random factors. Solid red lines represent the significant effects of TP. Vertical dashed lines separate the TP gradient into eutrophic (40–100 µg L^{-1}), hypereutrophic (100–300 µg L^{-1}), and highly hypereutrophic (>300 µg L^{-1}) levels. ns non-significant, * p<0.05, ** p<0.01, *** p<0.001.

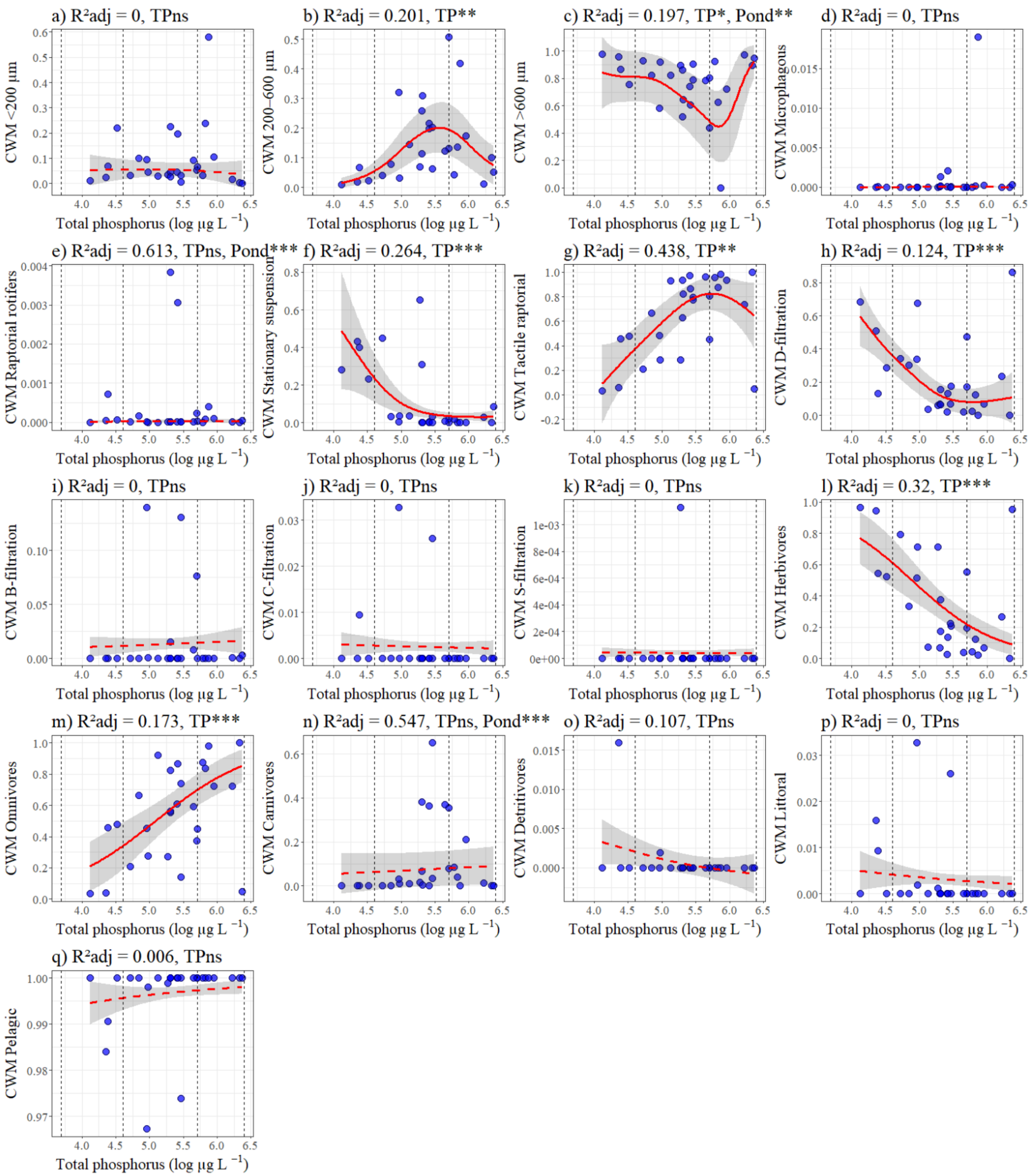

Figure S5 | Observed community-weighted means (CWM) of 17 zooplankton functional trait categories along the eutrophication gradient (log-transformed total phosphorus, TP). Models were fitted using generalized additive mixed models (GAMM), accounting for spatial and temporal autocorrelation by adding pond location and sampling time (month) as random factors. Solid red lines represent the significant effects of TP. Vertical dashed lines separate the TP gradient into eutrophic (40–100 μg L^{-1}), hypereutrophic (100–300 μg L^{-1}), and highly hypereutrophic (>300 μg L^{-1}) levels. ns non-significant, * p<0.05, ** p<0.01, *** p<0.001.

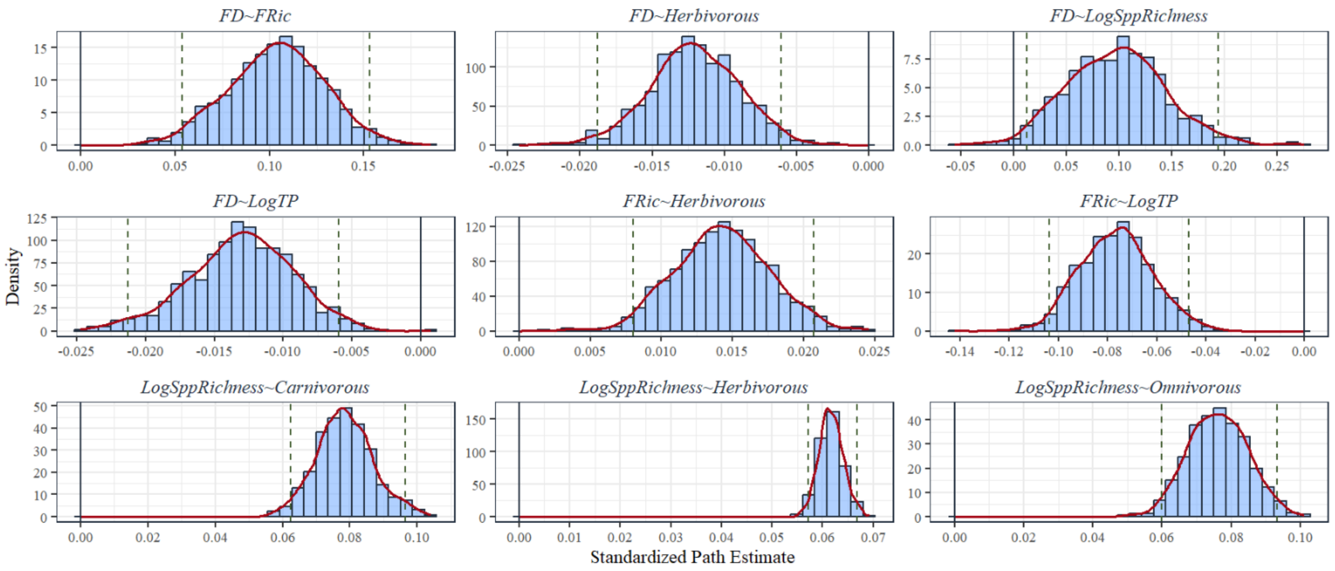

Figure S6 | Bootstrap distributions of standardized path estimates for each significant path in the piecewise structural equation model using the incidence data from March to September 2024 (n = 63, Figure 5a). Standardized path coefficients were obtained from 999 bootstrap resamplings. Dashed vertical lines indicate the lower and upper bounds of the 95% empirical confidence intervals for each path estimate, while the solid vertical line marks zero (no effect). Note that the zero line does not fall between the 95% confidence interval bounds in any of the paths, meaning that the SEM model properly estimated them.

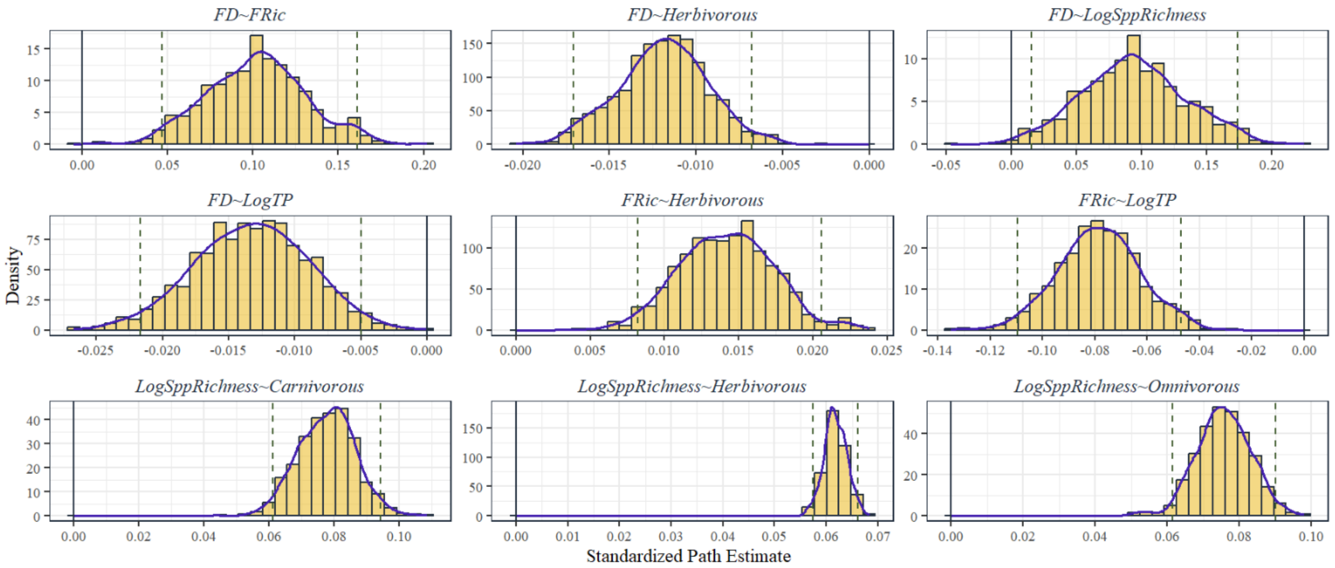

Figure S7 | Distribution of standardized path estimates from Monte Carlo power simulations of the piecewise SEM model using the incidence data from March to September 2024 (N = 63, B = 999, Figure 5a). Density plots and histograms show the variability in simulated path coefficients; dashed vertical lines represent 95% confidence intervals, and the solid vertical line marks zero (no effect). Note that the zero line does not fall between the 95% confidence interval bounds in any of the paths, meaning that the sample size was sufficient to test for the null hypotheses.

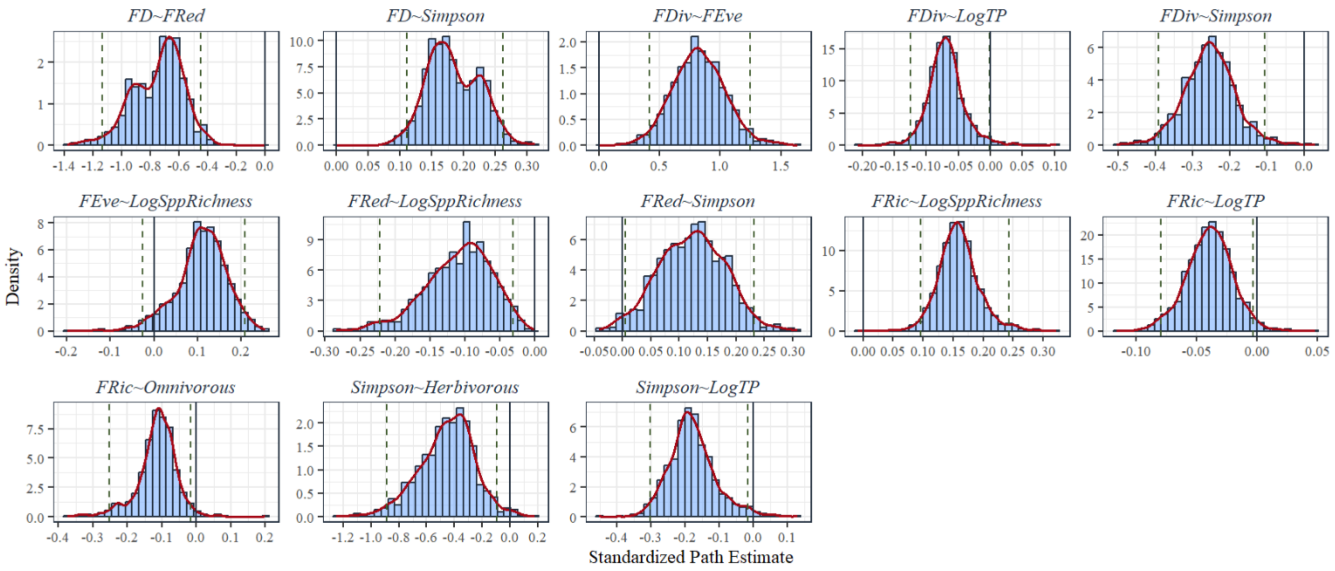

Figure S8 | Bootstrap distributions of standardized path estimates for each significant path in the piecewise structural equation model using the biomass data from July to September 2024 (n = 27, Figure 5b). Standardized path coefficients were obtained from 999 bootstrap resamplings. Dashed vertical lines indicate the lower and upper bounds of the 95% empirical confidence intervals for each path estimate, while the solid vertical line marks zero (no effect). Note that the zero line falls close to the lower 95% confidence interval bound for the path "*FEve~LogSppRichness*", which was removed from Figure 5b. The zero line does not fall between the 95% confidence interval bounds in any of the other paths, meaning that the SEM model properly estimated them.

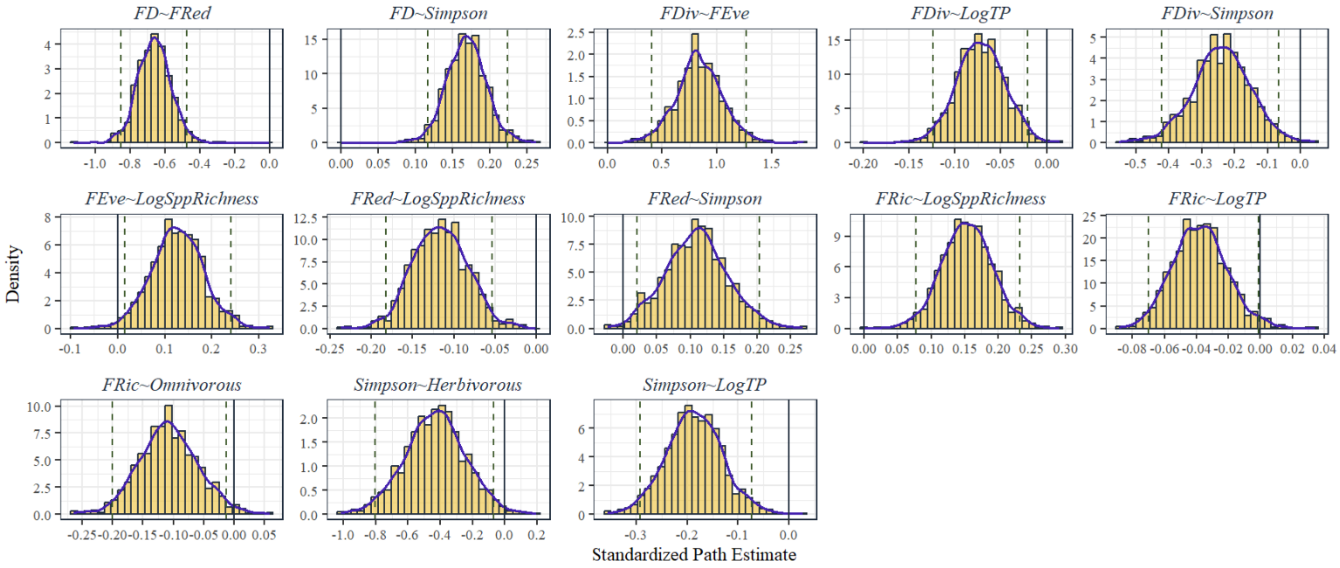

Figure S9 | Distribution of standardized path estimates from Monte Carlo power simulations of the piecewise SEM model using the biomass data from July to September 2024 (N = 27, B = 999, Figure 5a). Density plots and histograms show the variability in simulated path coefficients; dashed vertical lines represent 95% confidence intervals, and the solid vertical line marks zero (no effect). Note that the zero line does not fall between the 95% confidence interval bounds in any of the paths, meaning that the sample size was sufficient to test for the null hypotheses.

Table S1 | Output of generalized additive mixed models (GAMM) to test the effects of eutrophication (log-transformed TP, fixed factor) on observed zooplankton biomass, taxonomic diversity, functional diversity, and community-weighted means (CMW) of traits. The models account for spatial and temporal autocorrelation by adding pond location and sampling time (month) as random factors. Bold cells indicate significant effects.

	AIC	Adjusted R^2	F logTP	p logTP	F Pond	p Pond	F Month	p Month
Species richness	175.704	0.312	2.689	0.476	0.000	0.726	10.908	**0.010**
Simpson's diversity	-14.675	0.456	20.667	**<0.001**	0.000	0.384	0.000	0.800
Biomass (log-transformed)	42.746	0.800	3.534	0.077	12.056	**<0.001**	1.171	0.153
FRic (incidence)	-61.895	0.168	10.988	**<0.001**	1.604	0.281	2.561	0.184
FDis (incidence)	-118.528	0.219	18.589	**<0.001**	0.000	0.578	0.000	0.533
RaoQ (incidence)	-152.297	0.270	24.542	**<0.001**	0.000	0.662	0.000	0.589
FRic (biomass)	-42.108	0.364	8.415	**0.009**	1.122	0.073	0.000	0.446
FDis (biomass)	-36.541	0.304	13.441	**0.003**	0.000	0.824	0.000	0.528
RaoQ (biomass)	-58.854	0.313	5.287	**0.009**	0.000	0.608	0.000	0.476
FEve (biomass)	-32.689	0.447	4.521	**0.047**	2.310	**0.015**	0.000	0.582
FDiv (biomass)	-31.469	0.438	8.589	**0.021**	5.308	0.097	2.229	0.109
FRed (biomass)	-41.324	0.038	0.355	0.552	0.000	0.882	2.317	0.101
CWM <200 µm	-21.752	-0.049	0.151	0.779	0.203	0.237	0.364	0.223
CWM 200–600 µm	-29.678	0.201	6.846	**0.001**	0.065	0.442	0.000	0.872
CWM >600 µm	-10.513	0.197	10.842	**0.038**	14.267	**0.004**	0.509	0.266
CWM Microphagous	-218.622	0.099	2.707	0.079	14.227	**<0.001**	1.279	0.117
CWM Raptorial rotifers	-246.470	0.613	0.438	0.516	14.416	**<0.001**	0.000	0.506
CWM Stationary suspension	-137.993	0.264	27.427	**<0.001**	0.003	0.449	0.000	0.831
CWM Tactile-raptorial	4.436	0.438	6.482	**0.002**	0.000	0.561	0.000	0.588
CWM D-filtration	-3.161	0.124	30.173	**<0.001**	0.000	0.918	0.000	0.387
CWM B-filtration	-288.558	-0.044	0.359	0.549	0.000	0.824	0.000	0.691
CWM C-filtration	-354.389	-0.033	0.189	0.664	0.000	0.982	0.000	0.948
CWM S-filtration	-393.144	-0.040	0.001	0.980	0.000	1.000	0.000	0.925
CWM Herbivores	-15.487	0.320	20.074	**<0.001**	0.000	0.925	0.000	0.763
CWM Carnivores	-14.283	0.547	0.586	0.444	146.056	**<0.001**	0.000	0.495
CWM Omnivores	-8.894	0.173	14.685	**<0.001**	0.000	0.578	0.000	0.462
CWM Detritivores	-106.980	0.107	2.855	0.135	0.024	0.437	0.000	0.908
CWM Littoral	-273.807	0.000	1.308	0.253	0.000	0.950	0.000	0.673
CWM Pelagic	-309.401	0.006	1.931	0.165	0.000	0.831	0.000	0.528

Table S2 | Output of generalized additive mixed models (GAMM) to test the effects of eutrophication (log-transformed TP, fixed factor) on the standardized effect sizes (SES) for functional diversity and community-weighted means (CMW) of traits. The models account for spatial and temporal autocorrelation by adding pond location and sampling time (month) as random factors. Bold cells indicate significant effects.

	AIC	Adjusted R^2	F logTP	p logTP	F Pond	p Pond	F Month	p Month
SES FRic (incidence)	35.207	0.213	3.494	**0.019**	0.096	0.366	0.568	0.157
SES FDis (incidence)	92.787	0.216	18.087	**<0.001**	0.000	0.573	0.000	0.494
SES RaoQ (incidence)	87.382	0.265	23.394	**<0.001**	0.000	0.656	0.000	0.529
SES FRic (biomass)	76.427	0.396	8.565	**0.003**	13.012	**0.005**	0.000	0.312
SES FDis (biomass)	38.687	0.308	12.074	**0.004**	0.000	0.724	0.000	0.415
SES RaoQ (biomass)	37.431	0.317	5.240	**0.009**	0.000	0.603	0.000	0.459
SES FEve (biomass)	35.934	0.464	4.969	**0.038**	2.461	**0.012**	0.000	0.534
SES FDiv (biomass)	46.300	0.413	7.939	**0.019**	4.363	0.128	2.769	0.081
SES FRed (biomass)	37.431	0.317	5.240	**0.009**	0.000	0.603	0.000	0.459
SES <200 μm	31.654	-0.047	0.177	0.759	0.020	0.250	0.345	0.227
SES 200–600 μm	31.825	0.183	8.979	**0.035**	0.000	0.451	0.000	0.966
SES >600 μm	35.866	0.078	6.429	0.085	11.102	**0.006**	0.000	0.767
SES Microphagous	-12.532	-0.051	0.307	0.580	0.404	0.274	0.000	0.296
SES Raptorial rotifers	1.509	-0.046	0.991	0.490	0.000	0.547	0.000	0.666
SES Stationary suspension	36.859	0.481	5.529	**0.029**	1.236	0.081	0.000	0.426
SES Tactile-raptorial	40.291	0.321	29.933	**<0.001**	5.196	0.058	0.000	0.899
SES D-filtration	36.536	0.130	29.235	**<0.001**	0.000	0.899	0.000	0.378
SES B-filtration	6.022	-0.040	0.103	0.748	0.000	0.579	0.000	0.702
SES C-filtration	-13.566	-0.051	0.005	0.943	0.000	0.716	0.409	0.247
SES S-filtration	-42.853	-0.089	3.393	0.226	0.000	0.573	0.000	0.584
SES Herbivores	40.300	0.322	29.910	**<0.001**	5.253	0.056	0.000	0.898
SES Carnivores	33.362	0.541	0.556	0.456	133.399	**<0.001**	0.000	0.404
SES Omnivores	41.002	0.175	14.779	**<0.001**	5.191	0.063	0.000	0.614
SES Detritivores	-31.405	-0.057	1.282	0.560	0.000	0.513	0.000	0.551
SES Littoral	3.013	-0.034	0.470	0.493	0.000	0.953	0.000	0.897
SES Pelagic	3.013	-0.034	0.470	0.493	0.000	0.953	0.000	0.897

Table S3 | T-tests (T) or Wilcoxon tests (W) to examine if the SES values of functional diversity and community-weighted means (CMW) of traits are significantly different from zero, for the entire dataset or the trophic states (eutrophic: 40–100 µg L^{-1}; hypereutrophic: 100–300 µg L^{-1}; and highly hypereutrophic: >300 µg L^{-1}) levels. Bold cells indicate values significantly different from zero.

	Entire dataset	Eutrophic	Hypereutrophic	Highly hypereutrophic
SES FRic (incidence)	T= 0.82, p=0.413	**T= 2.73, p=0.013**	T= –0.03, p=0.973	T= –1.35, p=0.21
SES FDis (incidence)	T= –0.64, p=0.528	**T= 2.58, p=0.018**	T= –1.48, p=0.148	W=16, p=0.275
SES RaoQ (incidence)	T= –0.63, p=0.533	**T= 3.07, p=0.006**	T= –1.73, p=0.093	T= –2.25, p=0.051
SES FRic (biomass)	**W=377, p<0.001**	**T= 5.12, p=0.014**	**W=120, p<0.001**	**T= 2.77, p=0.028**
SES FEve (biomass)	T= –0.05, p=0.959	T= 1.16, p=0.33	T= 0.04, p=0.972	T= –0.76, p=0.471
SES FDiv (biomass)	**T= –3.72, p=0.001**	T= 2.71, p=0.073	**T= –4.98, p<0.001**	T= –2.16, p=0.068
SES FDis (biomass)	T= –1.82, p=0.08	T= 0.15, p=0.893	T= 0.06, p=0.952	**T= –3.24, p=0.014**
SES RaoQ (biomass)	T= –1.9, p=0.069	T= 0.48, p=0.667	T= –0.25, p=0.809	**W=0, p=0.008**
SES FRed (biomass)	T= 1.9, p=0.069	T= –0.48, p=0.667	T= 0.25, p=0.809	**W=36, p=0.008**
SES <200 µm	W=124, p=0.123	T= –0.44, p=0.689	W=28, p=0.073	W=16, p=0.844
SES 200–600 µm	W=119, p=0.095	W=0, p=0.125	T= 0.21, p=0.837	T= –0.89, p=0.401
SES >600 µm	W=263, p=0.077	**T= 3.2, p=0.049**	T= 0.62, p=0.545	W=24, p=0.461
SES Microphagous	**W=51, p<0.001**	**T= –11.57, p=0.001**	**W=13, p=0.005**	W=8, p=0.195
SES Raptorial rotifers	**W=64, p=0.002**	W=3, p=0.625	W=29, p=0.083	**T= –8.63, p<0.001**
SES Stationary suspension	W=168, p=0.628	**T= 4.42, p=0.022**	W=42, p=0.33	**W=0, p=0.008**
SES Tactile-raptorial	W=187, p=0.972	**T= –3.22, p=0.049**	T= 0.1, p=0.921	W=28, p=0.195
SES D-filtration	W=163, p=0.546	T= 1.62, p=0.203	T= –0.21, p=0.836	W=9, p=0.25
SES B-filtration	**W=78, p=0.006**	**T= –64.2, p<0.001**	W=42, p=0.33	**T= –34.18, p<0.001**
SES C-filtration	**W=78, p=0.006**	W=4, p=0.875	W=29, p=0.083	**T= –34.77, p<0.001**
SES S-filtration	**W=27, p<0.001**	**T= –21.58, p<0.001**	**W=15, p=0.008**	**T= –36.77, p<0.001**
SES Herbivores	W=191, p=0.972	**T= 3.22, p=0.049**	T= –0.1, p=0.921	W=8, p=0.195
SES Carnivores	W=147, p=0.324	**T= –105.96, p<0.001**	W=54, p=0.762	W=15, p=0.742
SES Omnivores	T= –0.36, p=0.724	T= –2.48, p=0.089	T= –0.42, p=0.682	T= 1.22, p=0.261
SES Detritivores	**W=53, p=0.001**	W=4, p=0.875	**W=15, p=0.008**	**T= –25.8, p<0.001**
SES Littoral	**W=102, p=0.036**	T= 0.53, p=0.634	W=29, p=0.083	**T= –34.82, p<0.001**
SES Pelagic	**W=276, p=0.036**	T= –0.53, p=0.634	W=91, p=0.083	**T= 34.82, p<0.001**
